# Silk-Ovarioids: establishment and characterization of a human ovarian primary cell 3D-model system

**DOI:** 10.1093/hropen/hoaf042

**Published:** 2025-07-10

**Authors:** Valentina Di Nisio, Tianyi Li, Zhijie Xiao, Kiriaki Papaikonomou, Anastasios Damdimopoulos, Ákos Végvári, Filipa Lebre, Ernesto Alfaro-Moreno, Mikael Pedersen, Terje Svingen, Roman Zubarev, Ganesh Acharya, Pauliina Damdimopoulou, Andres Salumets

**Affiliations:** Department of Gynecology and Reproductive Medicine, Karolinska University Hospital, Huddinge, Stockholm, Sweden; Department of Women’s and Children’s Health, Karolinska Institutet, Stockholm, Sweden; Celvia CC, Competence Centre On Health Technologies, Tartu, Estonia; Department of Oncology and Pathology, Karolinska Institutet, Stockholm, Sweden; BioLamina AB, Stockholm, Sweden; Department of Gynecology and Reproductive Medicine, Karolinska University Hospital, Huddinge, Stockholm, Sweden; Department of Women’s and Children’s Health, Karolinska Institutet, Stockholm, Sweden; Bioinformatics and Expression Analysis Core Facility, Karolinska Institutet, Huddinge, Stockholm, Sweden; Division of Chemistry I, Department of Medical Biochemistry and Biophysics, Karolinska Institutet, Stockholm, Sweden; NanoSafety Group, International Iberian Nanotechnology Laboratory, Braga, Portugal; NanoSafety Group, International Iberian Nanotechnology Laboratory, Braga, Portugal; National Food Institute, Technical University of Denmark, Kongens Lyngby, Denmark; National Food Institute, Technical University of Denmark, Kongens Lyngby, Denmark; Division of Chemistry I, Department of Medical Biochemistry and Biophysics, Karolinska Institutet, Stockholm, Sweden; Division of Obstetrics and Gynecology, Department of Clinical Science, Intervention and Technology, Karolinska Institutet, Huddinge, Sweden; Department of Obstetrics, Center for Fetal Medicine, Karolinska University Hospital, Stockholm, Sweden; Department of Gynecology and Reproductive Medicine, Karolinska University Hospital, Huddinge, Stockholm, Sweden; Department of Women’s and Children’s Health, Karolinska Institutet, Stockholm, Sweden; Department of Gynecology and Reproductive Medicine, Karolinska University Hospital, Huddinge, Stockholm, Sweden; Celvia CC, Competence Centre On Health Technologies, Tartu, Estonia; Division of Obstetrics and Gynecology, Department of Clinical Science, Intervention and Technology, Karolinska Institutet, Huddinge, Sweden; Department of Obstetrics and Gynaecology, Institute of Clinical Medicine, University of Tartu, Tartu, Estonia

**Keywords:** human ovary, primary cells, 3D model, Biosilk, long-term culture

## Abstract

**STUDY QUESTION:**

What is the best protocol to establish a long-term stable three-dimensional (3D) model for human primary ovarian cells?

**SUMMARY ANSWER:**

We developed and characterized long-term cultured 3D models of primary ovarian somatic cells isolated from adult tissues, using Biosilk as a scaffold.

**WHAT IS KNOWN ALREADY:**

*In vitro* models that mimic ovaries are crucial for elucidating the biological mechanisms underlying follicle activation and growth, hormonal activity, ovarian angiogenesis, damage in response to toxic exposures, and other biological mechanisms that enable the functionality of this complex organ. Three-dimensional systems are particularly relevant because they replicate heterogeneity and cell–cell communication among different ovarian cell types. However, complex models using human ovarian primary cells are yet to be developed.

**STUDY DESIGN, SIZE, DURATION:**

Ovarian tissue samples were collected from five patients (age 26 ± 5 years) who underwent gender-affirming surgery. The cortex and medulla were separated and dissociated into single-cell suspensions using mechanical and enzymatic methods. Three approaches were tested to establish a 3D model culture system: matrix-free ovarian spheroids (MFOS), a Matrigel-based three-layer gradient system (3LGS), and Biosilk scaffolds (Silk-Ovarioid). In parallel, paired controls from each patient and ovarian area were cultured in a standard 2D system for the same duration.

**PARTICIPANTS/MATERIALS, SETTING, METHODS:**

The 3D culture systems were monitored every second day to detect signs of aggregation and growth. Freshly fixed tissue, as well as 2D- and 3D-cultured samples were further processed for transcriptomic profiling after 42 days of culture using RNA sequencing. The culture of the 3D system was further characterized, regarding its protein profile and steroid and cytokine production, through proteomics and liquid chromatography–tandem mass spectrometry and the Luminex platform, respectively. The key findings from the high-throughput assays were finally validated through RNA fluorescent *in situ* hybridization (RNA-FISH) and immunofluorescence staining.

**MAIN RESULTS AND THE ROLE OF CHANCE:**

The 3D model systems MFOS (n = 120) and 3LGS (n = 18) failed to form aggregates capable of long-term maintenance in culture (MFOS: maximum of 15 days for both cortex and medulla; 3LGS: maximum of 11 days for medulla only). In contrast, we successfully established ovarian cortex- and medulla-derived 3D systems using Biosilk, termed Silk-Ovarioids (n = 120). Silk-Ovarioids were maintained for up to 42 days as free-floating culture without any signs of cell death, as confirmed by the absence of TUNEL, γ-H2A.X, and cleaved caspase 3 fluorescent signals. The presence of key ovarian somatic cell types, including granulosa, stromal, endothelial, and perivascular cells, was confirmed by transcriptomics and proteomics in the majority of Silk-Ovarioids. Validation through RNA-FISH and immunostaining was performed using the following markers: AMHR2 for granulosa cells, PDGFRα for stromal cells, CLDN5 and GPIHBP1 for endothelial cells, GJA4/Cx37 and MCAM for perivascular cells. Notably, Silk-Ovarioids exhibited the formation of a pro-angiogenic hypoxic core, as evidenced by the transcriptomic and proteomic data and visualized by the expression of hypoxia markers MMP2 and PDGFRβ. This hypoxic environment led to development of vessel-like structures after 4–6 weeks of culture, which were positive for the angiogenic markers TGFBR2, BMP2, and PDGFα. The functionality of Silk-Ovarioids was further confirmed by the identification of *de novo* extracellular matrix secretion (Col1α1 and Lamα1), and by the detection of pro-angiogenic cytokines (e.g. IL-6, IL-8, and GM-CSF) and steroids (e.g. pregnenolone and epitestosterone) in the culture media.

**LARGE SCALE DATA:**

The RNA-sequencing count matrix is deposited in Gene Expression Omnibus with accession number GSE253571. Raw data are deposited in Swedish National Data Service with the DOI https://doi.org/10.48723/h8cm-bs19. Single-cell RNA-seq data have been downloaded from the ArrayExpress database at EMBL-EBI with the accession codes ‘E-MTAb − 8381’. The mass spectrometry proteomics data have been deposited to the ProteomeXchange Consortium via the PRIDE partner repository with the dataset identifier PXD048710. The code used for the analysis can be found in https://github.com/tialiv/Silk-Ovarioid_project.

**LIMITATIONS, REASONS FOR CAUTION:**

The ovarian samples were collected from patients undergoing androgen treatment, raising the concern that androgen exposure may alter the behavior of cells in Silk-Ovarioids compared to those derived from androgen-unstimulated patients. Furthermore, the cell culture media used in this study were supplemented with fetal bovine serum and did not contain any supplements or growth factors that could be essential for the resemblance of Silk-Ovarioids to the tissue of origin.

**WIDER IMPLICATIONS OF THE FINDINGS:**

The Silk-Ovarioids exhibited low intra-batch variability and long-term culture stability, underscoring their potential as a robust step toward developing a bioengineered, patient-specific artificial ovary. In addition, Silk-Ovarioids could be utilized as the first ovarian angiogenesis *in vitro* model, function as biological scaffold for *in vitro* folliculogenesis, and be used for toxicological and pharmacological studies targeting the ovaries.

**STUDY FUNDING/COMPETING INTEREST(S):**

This study was funded by: a research grant from the Center for Innovative Medicine (CIMED) at Karolinska Insitutet; European Union’s Horizon 2020 Research and Innovation Programme (project ERIN no. 952516); a Horizon Europe grant (NESTOR, grant no. 101120075) of the European Commission; the Swedish Research Council for Sustainable Development FORMAS (2018-02280, 2020-01621); StratRegen Funding from Karolinska Institute, Swedish Research Council VR (grant no. 2020-02132); Swedish Childhood Cancer Fund (Reference PR2017-0044, PR2020-0096); Estonian Research Council (grant no. PRG1076); Swedish Research Council (grant no. 2024-02530); Novo Nordisk Foundation (grant no. NNF24OC0092384); European Union’s H2020 project Sinfonia (no. 857253) (INL research); and SbDToolBox, with reference NORTE-01-0145-FEDER-000047, supported by Norte Portugal Regional Operational Programme (NORTE 2020), under the PORTUGAL 2020 Partnership Agreement, through the European Regional Development Fund (INL research). The authors have no conflicts of interest to declare.

WHAT DOES THIS MEAN FOR PATIENTS? Understanding how ovaries function is essential for studying fertility, ovarian diseases, and reproductive toxicity, and for developing or testing new treatments. Traditional 2D cell culture methods are insufficient in capturing complex interactions among different ovarian cell types. While 3D models have been attempted, they have not yet been shown to support long-term survival and growth of human ovarian cells.

To identify the best approach, we tested three different methods using ovarian cells from patients. We successfully developed a new 3D model using a material called Biosilk that allows long-term growth of human ovarian cells in the laboratory; the method has been named Silk-Ovarioids. Silk-Ovarioids succeeded in allowing the cells to grow and survive for over 6 weeks. On the contrary, the other methods failed within 2 weeks. Silk-Ovarioids contain the main cell types of the ovary, including cells that support oocyte development and others promoting blood vessel growth. The Silk-Ovarioids even formed small, blood vessel-like structures and produced essential hormones and proteins.

This discovery could significantly advance research in reproductive health. Silk-Ovarioids provide a powerful method for studying ovarian diseases, for testing new drugs and toxicity of chemicals, and, potentially, for creating artificial ovaries for patients in the future. This discovery may contribute to improved fertility treatments, personalized medicine, and better ways to study and manage ovarian health issues. While more work is needed, this is a promising step toward advancing reproductive healthcare.

## Introduction

A woman’s fertility potential depends on the health of her reproductive organs, including ovaries and the ovarian reserve (La Marca *et al.*, 2012). The ovarian reserve is defined as the number of dormant follicles in the ovarian cortex that can either be stimulated to grow and mature or undergo atresia. The decline of ovarian reserve can be due to natural and physiological factors (e.g. age), pathological conditions (e.g. premature ovarian insufficiency, POI), or exposure to toxic chemicals or pharmaceuticals (e.g. iatrogenic POI) (Vabre *et al.*, 2017; Mehedintu *et al.*, 2021; Bai and Wang, 2022; Esencan *et al.*, 2022). Improvements in the treatment of childhood and young adult cancers and hematological diseases have led to increased survival rates. Consequently, the number of survivors living with long-term side effects, like infertility and POI, is rising (Donnez *et al.*, 2006).

From a clinical perspective, ovarian tissue cryopreservation for future auto-transplantation is currently the only procedure available for prepubertal girls at very high risk of infertility due to gonadotoxic treatment (Donnez *et al.*, 2006; Antonouli *et al.*, 2023). However, for diseases with systemic malignancies like leukemias, the high risk of reintroducing malignant cells constitutes a major concern and restricts the applicability of this approach (Diáz-Garciá *et al.*, 2019). These cases necessitate the exploration of alternative strategies, such as *in vitro* follicle growth and the collection of immature oocytes for further *in vitro* maturation in ART laboratories.

To create a better three-dimensional (3D) environment for *in vitro* follicle growth, the presence of main functional ovarian somatic cell populations is pivotal. For instance, a common requirement in ovarian 3D systems is the introduction or induction of extracellular matrix (ECM) formation in culture (Hovatta *et al.*, 1997; Shikanov *et al.*, 2011; Desai *et al.*, 2012). ECM is crucial for the survival, growth, and structural organization of somatic cells, which are essential for supporting *in vitro* follicle development in mammals (Grosbois *et al.*, 2023). As reported in previous studies, the human ovarian tissue architecture is based on the expression of laminin, collagen, and fibronectin, which overall support the different ovarian functions during the female reproductive lifespan (Hao *et al.*, 2020; Ouni *et al.*, 2021, 2022). Despite the advancements of *in vitro* ovarian cortical tissue culture (Hao *et al.*, 2024), this system has many limitations that make this gold standard insufficient for the next steps of *in vitro* ovarian studies on both follicles and somatic compartments. Other ovarian 3D models as well have many drawbacks, mainly concerning the short culture period, the lacking in protocol standardization, and the use of animal models in the establishment of the system, making them difficult to translate to humans (Chen *et al.*, 2025). Examples of ongoing approaches for the development of different 3D ovarian *in vitro* models include bioprinted, gelatin-based scaffolds (Laronda *et al.*, 2017), and decellularized ovarian tissue, as support structures for the reintroduction of ovarian somatic cells and follicles (Pors *et al.*, 2019; Alshaikh *et al.*, 2020; Chiti *et al.*, 2022). In addition, currently available ovarian 3D models are not optimal for the distribution of oxygen and nutrients to the inner core of the structure, which could be improved by incorporating the somatic cells that are able to recreate capillaries and small vessels (*de novo* angiogenesis) in the systems (Hao *et al.*, 2020; Chen *et al.*, 2025). Therefore, there is a need to explore new and diverse 3D models to overcome this bottleneck and enhance ovarian culture systems for both somatic and germ cells research.

Additionally, new technologies have been applied in *in vitro* ovarian modeling to study physio-pathological conditions, such as ovarian cancer. These technologies include the development of organ-on-a-chip systems using microfluidic devices (Yan *et al.*, 2023) and the reconstruction of ovaries from pluripotent stem cells in mice (Yoshino *et al.*, 2021). Apart from that, human-specific ovarian organoids represent a promising alternative to animal models, with the added benefit of exploring personalized treatments using patient-specific models (Nanki *et al.*, 2020; Graham *et al.*, 2023). Even though newer materials (e.g. polyethylene glycol (PEG)ylated fibrin hydrogels) are now being tested for their abilities to support human ovarian somatic cells and follicles growth (Dadashzadeh *et al.*, 2021, 2023), Matrigel is still the most used matrix for stem cell-derived organoids culture in diverse cell types (Kyriakopoulou *et al.*, 2023). As an example, Matrigel has been used to develop the three-layer gradient system (3LGS) and leads to formation of testicular and ovarian organoids from human fetal gonads (Oliver *et al.*, 2021). Nevertheless, we still lack *in vitro* models derived from healthy ovarian tissue that can be maintained in long-term culture and used to advance our understanding of ovarian biology and function. Major barriers to this progress include the limited access to human ovarian tissues needed for model development, as well as the difficulties in handling and cultivating ovarian cells.

To address these challenges and identify the most suitable protocol for long-term culture of human primary ovarian somatic cells, we aimed to investigate 3D models obtained through diverse approaches. We tested three distinct methodologies using ovarian cells dissociated from the same patient samples: matrix-free ovarian spheroids (MFOS), a Matrigel-based 3LGS, and a Biosilk-based floating culture system (Silk-Ovarioids). Somatic cells from adult patients grew robustly only in the Biosilk system, where they formed Silk-Ovarioids that could be maintained in a floating culture for up to 6 weeks. Meeting our goal of culturing a heterogeneous ovarian cell population, Silk-Ovarioids harbored all key ovarian somatic cell types and exhibited pro-angiogenic activity.

## Materials and methods

### Ethics statement

The use of ovarian tissue in this research was approved by the Stockholm Region Ethical Review Board (Dnr. 2015/798–31/2 with amendments). Clinicians informed the patients about the study, and ovarian tissue was collected from patients who underwent gender-affirming surgery after written and signed informed consent was obtained.

### Ovarian tissue handling and dissociation into single-cell suspensions

Ovarian tissue samples were collected from five patients (age 26 ± 5 years) who underwent gender-affirming surgery at Karolinska University Hospital and signed informed consent for ovarian tissue donation and medical records data collection. Patients’ data handling was performed in accordance with the European General Data Protection Regulation guidelines. All samples were pseudonymized using random codes. On the day of the surgery, samples were transported from the operating room to the laboratory in Dulbecco’s phosphate-buffered saline containing calcium, magnesium, glucose, and pyruvate (Thermo Fisher Scientific, Grand Island, NY, USA). Upon sample receival, the medulla was separated from the cortex before tissue dissociation.

Dissociation of cortex and medulla was performed following our previously published protocol (Wagner *et al.*, 2020). In summary, the cortex and medulla were cut into pieces smaller than 1 mm^3^ and placed in digestion medium consisting of DMEM/F12 (Thermo Fisher Scientific), 2.5% heat-inactivated fetal bovine serum (HI-FBS, Gibco, Life Technologies, Grand Island, NY, USA), 1 mg/ml collagenase IA (Sigma Aldrich, Saint Louis, MO, USA), 50 μg/ml Liberase™ (Roche Diagnostics, Mannheim, Germany), and 10 IU/ml DNase I (Roche Diagnostics). The trimmed pieces were then dissociated for a maximum of 50 min in a shaking water bath at 37°C, and the reaction was terminated with an equal volume of termination medium (i.e. DMEM/F12 supplemented with 10% HI-FBS). Subsequently, cell suspensions were centrifuged at 300 *g* for 5 min, followed by resuspension in culture medium (DMEM low glucose (Thermo Fisher Scientific), 10% HI-FBS, 1% Penicillin/Streptomycin (Life Technologies)), and filtered through a 40 μm cell strainer (VWR, Radnor, PA, USA). The obtained single-cell suspensions of ovarian primary cells were seeded either for monolayer 2D culture (1.5 × 10^6^ cells/well) in 6-well plates (Sarstedt, Numbrecht, Germany) or 3D culture, as outlined below. In all culture systems, half of the culture medium was refreshed every second day. Pictures of the aggregates were taken at every media change using an inverted microscope (Olympus CKX41, Carl Zeiss Meditec, Germany).

### 3D systems for primary ovarian cell culture

#### Matrix-free ovarian spheroids

After dissociation, ovarian primary cells were seeded at different densities (3 × 10^4^, 6 × 10^4^, 1.2 × 10^5^ cells/well) in 96-well ultra-low attachment plates (Corning, Corning, NY, USA). Cells were left undisturbed for 2 days after seeding, to allow aggregation without disrupting the culture microenvironment. From the third day onward, half of the culture media was changed every second day.

#### Matrigel-based 3LGS

For the Matrigel-based spheroids formation, the methodology for 3LGS as described by Alves-Lopes *et al.* (2018) was followed. Briefly, Matrigel (1:1; Corning) was diluted in ice-cold culture medium to make the first 5 µl base drop onto the hanging cell insert (PIHT12R48, Millipore, Darmstadt, Germany). After the solidification of the first drop in the incubator, the primary ovarian cell suspension was diluted in 3 µl of Matrigel-culture medium (1.2 × 10^5^ cells/drop) and carefully placed on top of the base drop. The Matrigel-embedded cell suspension drop was left to solidify in the incubator for 15 min. Lastly, the embedding Matrigel drop of 8 µl was placed on top of the existing ones, to ensure the complete covering. After solidification of the last drop for 20 min in the incubator, the hanging cell insert was carefully placed in a 24-well plate (Sarstedt, Numbrecht, Germany) and submerged in 600 µl of pre-warmed culture medium. Half of the culture medium was changed every second day.

#### Silk-based 3D culture: Silk-Ovarioids

Biosilk™ protein solution (No. BS-0101, BioLamina, Sundbyberg, Sweden) was used to create the scaffolds for single-cell seeding according to the manufacturer’s instructions. The Biosilk™ fibers mainly consist of recombinant spindroins, a spider silk protein, further biofunctionalized with arginylglycylaspartic acid (RGD)-containing motif sequence. Briefly, a 20 µl drop of Biosilk™ was placed in the center of a 24-well plate (Sarstedt), and air bubbles were introduced by pipetting up and down (20–22 times), followed by the establishment of a dense and compact foam of 1 cm diameter maximum. A total of 12 foams were generated in the series. Ovarian primary cells dissociated from the cortex and medulla were prepared as separate single-cell suspensions and seeded at the chosen density (1.2 × 10^5^ cells/foam, with six foams using cortex and six foams using medulla-derived ovarian primary cells). The cell suspension was added to the silk foam and mixed into a homogeneous solution by pipetting 5–6 times. The seeded cells were left to stabilize on the foam in the incubator for 20 min. After stabilization, pre-warmed culture medium was carefully added drop-by-drop to the cell–foam mixture. The seeded foams were left untouched for 3 days, after which half of the medium was changed every second day. After 14 days, the seeded foams were lifted from the well using a spatula and divided into two halves. Each separated half-foam (from now on referred to as Silk-Ovarioids) was then transferred into a flat-bottom ultra-low attachment 24-well plate (Corning) and kept in free-floating culture for 30–42 days. Half of the medium was changed every second day and collected every 7 days for further analysis. At the end of the culture period, Silk-Ovarioids were harvested in RNALater^®^ (Invitrogen, Vilnius, Lithuania) and snap frozen at −80°C for further analysis, and in 4% methanol-free formaldehyde (Thermo Fisher Scientific). This process was performed for each patient-specific Silk-Ovarioids culture, with each considered as a separate individual batch.

### RNA extraction and library preparation from samples

RNA extraction from tissues stored in RNALater^®^ and 2D cultured cells (Day 42 of culture) lysed RLT buffer (Qiagen, Hilden, Germany) was performed using a RNeasy Mini Kit (Qiagen) according to the manufacturer’s instructions. In brief, tissues were transferred to a gentleMACS™ M tube (Miltenyi Biotec, Bergisch Gladbach, Germany) containing 350 µl of RLT buffer. Tissues were fully homogenized using the RNA_01.01 program provided in the gentleMACS™ Dissociator (Miltenyi Biotec). The lysate was then incubated with proteinase K (600 mAU/ml, diluted 1:60 in RNase-free water; Qiagen) to digest proteins. For 2D cultured cells, 350 µl of RLT buffer was used for cell harvest. Thereafter, the lysates from both tissues and 2D cultured cells were cleaned up before DNase I (Qiagen) treatment for residual DNA removal.

Total RNA from Silk-Ovarioids samples (Day 42 of culture) was extracted using a RNeasy Micro Kit (Qiagen) according to the manufacturer’s instructions. In summary, harvested Silk-Ovarioids were resuspended in 75 µl of RLT buffer, lysed mechanically with an insulin syringe, and processed for extraction with DNase I (Qiagen) treatment. The yield of RNA was measured with a NanoPhotometer (IMPLEN, Nordic Biolabs, Taby, Sweden), with subsequent determination of quality (i.e. RNA integrity [RIN] value) and quantity using an Agilent Bioanalyzer 2100 (Agilent Technologies, Santa Clara, CA, USA). Libraries were prepared at the Bioinformatics and Expression Analysis (BEA) core facility at Karolinska Institute, Sweden, from samples with RIN values >9 and A260/A280 > 1.8, using the Illumina Stranded mRNA Prep Ligation protocol (Illumina, San Diego, CA, USA). Following the manufacturer’s protocol, we used 10 ng of RNA for library preparation. Subsequently, libraries were sequenced on the NovaSeq6000 platform at the National Genomics Infrastructure (NGI), SciLife Lab, Sweden.

### RNA sequencing and data analysis

STAR aligner (version 2.7.10b) was used to map the trimmed fastq files to the human genome GRCh38. Subsequently, the subread package (version 2.0.1) was used to align the bam files through *featureCount* function, and to annotate using Ensembl gtf file (Homo_sapiens.GRCh38.108.chr.gtf). The pipeline used for mapping and alignment was provided in a snakemake file. Differential expression analysis was performed in R (version 4.2.3) and Bioconductor through RStudio (RStudio Team, 2020; R Core Team, 2022) using the DESeq2 package. Technical replicates of the samples were collapsed using the *collapseReplicates* function. One Silk-Ovarioid sample from the cortex was removed due to low library size. Comparisons of Silk-Ovarioids versus tissue, 2D cell culture versus tissue, and Silk-Ovarioids versus 2D cell culture were performed separately for cortex and medulla datasets. To obtain differentially expressed genes (DEGs), cutoff of false discovery rate (FDR)<0.05, absolute log_2_-fold change (log_2_FC)>2, and average expression (baseMean)>100 were used. Affected biological pathways were identified using gene set enrichment analysis (GSEA) against MSigDB (Liberzon *et al.*, 2011) hallmark gene sets and gene ontology (GO) analysis through clusterProfiler (Yu *et al.*, 2012; Wu *et al.*, 2021), pathview (Luo and Brouwer, 2013), DOSE (Yu *et al.*, 2015), and the apeglm package (Zhu *et al.*, 2019). ComplexHeatmap package (Gu *et al.*, 2016; Gu, 2022) was used for heatmap plotting in RStudio.

Cell cycle-related genes were collected from the cell cycle scoring function in the Seurat package (Hao *et al.*, 2021). Genes involved in hypoxia and angiogenesis pathways were extracted from the GO database. Genes related to steroidogenesis were identified through literature review. Gene expression data were presented as the average scaled values from biological replicates within each group in the heatmap. The genes in the heatmap were clustered using k-means clustering to identify expression patterns. Subsequently, genes in each cluster were enriched using GO over-representation analysis to identify affected signaling pathways.

Deconvolution of RNA-seq data was performed using the dampened weighted least squares (DWLS) method (Tsoucas *et al.*, 2019). Count matrix was normalized to library size using the *cpm* function from the edgeR package. Subsequently, we downloaded the integrated single-cell RNA-seq data from ovarian cortex and medulla (Fan *et al.*, 2019; Wagner *et al.*, 2020) and manually annotated the data into five main clusters (i.e. stroma/theca, perivascular, endothelial, monocytes, and granulosa cells). Oocytes were not annotated separately as our *in vitro* culture systems did not contain this cell type. To obtain a non-biased signature matrix, we randomly selected 1000 cells from each cluster and used them for cell-type markers identification. The signature matrix was calculated using the *buildSignatureMatrixMAST* function based on the single-cell RNA-seq (scRNA-seq) reference. Deconvolution was performed using the *solveDampenedWLS* function.

Gene concept network plots related to hypoxia and angiogenesis gene sets were plotted using the enriched significant DEGs through the *cnetplot* function.

### Proteomics sample preparation

Tissue samples were supplemented with 12.5 µl of 8M urea, shaken vigorously, and sonicated in a water bath for 5 min before 50 µl of 0.2% ProteaseMAX (Promega, Madison, WI, USA) in 10% acetonitrile (ACN) and 100 mM Tris–HCl, pH 8.5 and 1 µl of 100× protease inhibitor (Roche Diagnostics) were added and mixed. Following this step, 36.5 µl of 50 mM Tris–HCl was added and the samples were sonicated again using a VibraCell probe (Sonics & Materials, Inc., Newton, CT, USA) for 40 s with an on/off pulse with a 2 s interval at 20% amplitude. Lysates were spun down at 13 000 *g* at 4°C for 10 min and protein concentration was determined by the Pierce™ BCA Protein assay kit (Thermo Fisher Scientific). A volume of lysate corresponding to 25 µg of protein was taken and supplemented with Tris–HCl buffer up to 100 µl. Proteins were reduced by adding 3.5 µl of 250 mM dithiothreitol (Sigma Aldrich) and incubated at 45°C for 37 min while shaking on a block heater. Alkylation was performed with addition of 4 µl of 500 mM iodoacetamide (Sigma Aldrich) at room temperature (RT) for 30 min in the dark. Then, 0.5 µg of sequencing-grade modified trypsin (Promega) was added to the samples and incubated for 16 h at 37°C. The digestion was stopped with 6 µl concentrated (cc.) formic acid (FA), incubating the solutions at RT for 5 min. The samples were cleaned on a C18 Hypersep plate with 40 µl bed volume (Thermo Fisher Scientific), and dried using a vacuum concentrator (Eppendorf, Leipzig, Germany).

Silk-Ovarioids were thawed on ice and lysed with addition of 10 µl of 8M urea and sonicated in a water bath for 5 min before 70 µl of 0.5M NaCl in 50 mM Tris–HCl, pH 8.5, and 0.8 µl 100× protease inhibitor (Roche Diagnostics) were added. Following sonication, the lysates were spun down at 13 000 *g* at 4°C for 10 min and protein concentration was determined by the Pierce™ BCA Protein assay kit (Thermo Fisher Scientific). A volume of lysate corresponding to 7.7 µg of protein was taken and supplemented with 1M urea and 438 mM NaCl in Tris–HCl buffer up to 75 µl. Proteins were reduced by adding 2.8 µl of 250 mM dithiothreitol (Sigma Aldrich) and incubated at 37°C for 45 min while shaking on a block heater. Alkylation was performed with addition of 3.1 µl of 500 mM iodoacetamide (Sigma Aldrich) at RT for 30 min in the dark. Proteolytic digestion was achieved by adding 0.4 µg sequencing grade modified trypsin (Promega) to the samples and incubating for 16 h at 37°C. The digestion was stopped with 4.5 µl cc. FA, incubating the solutions at RT for 5 min. The samples were cleaned on a C18 Hypersep plate with 40 µl bed volume (Thermo Fisher Scientific), and dried using a vacuum concentrator (Eppendorf).

Both tissue and Silk-Ovarioid samples were labeled with TMT-10plex (Thermo Fisher Scientific) isobaric reagents in two sets. Peptides were solubilized in 70 µl of 50 mM triethylammonium bicarbonate and mixed with 100 µg TMT-10plex reagents in anhydrous ACN and incubated for 2 h at RT. The unreacted reagents were quenched with 6 µl of hydroxyamine for 15 min at RT. Biological samples were then combined, dried in vacuum, and cleaned on a C18 Hypersep plate.

### Liquid chromatography–tandem mass spectrometry data acquisition and analysis

TMT-10plex labeled peptide samples were reconstituted in solvent A (2% ACN, 0.1% FA in water) and ∼2 µg of samples was injected onto a 50 cm long EASY-Spray C18 column (Thermo Fisher Scientific) connected to an Ultimate 3000 nanoUPLC system (Thermo Fisher Scientific) using a 90 min long gradient: 4–26% of solvent B (98% ACN, 0.1% FA) in 90 min, 26–95% in 5 min, and 95% of solvent B for 5 min at a flow rate of 300 nl/min. Mass spectra were acquired using a Q Exactive HF hybrid quadrupole-Orbitrap mass spectrometer (Thermo Fisher Scientific) in a range of m/z 375–1700 at a resolution of *R* = 120 000 (at m/z 200) targeting 1×10^6^ ions for a maximum injection time of 80 ms, followed by data-dependent higher-energy collisional dissociation (HCD) fragmentations of the top 18 precursor ions with a charge state 2+ to 7+, and using a 45 s dynamic exclusion. The tandem mass spectra were acquired at a resolution of *R* = 60 000, targeting 2×10^5^ ions for a maximum injection time of 54 ms, setting quadrupole isolation width to 1.4 Th and normalized collision energy to 34%.

Acquired raw data files were analyzed using Proteome Discoverer v3.0 (Thermo Fisher Scientific) with the MS Amanda v2.0 search engine against the human protein database (SwissProt, 20 022 entries downloaded on 9 February 2023). A maximum of two missed cleavage sites were allowed for full tryptic digestion, while setting the precursor and the fragment ion mass tolerance to 10 ppm and 0.02 Da, respectively. Carbamidomethylation of cysteine was specified as a fixed modification. Oxidation on methionine, deamidation of asparagine and glutamine, TMT6plex (+229.163 Da) of lysine, and peptide N-termini were set as dynamic modifications. Initial search results were filtered with 5% FDR using the Percolator node in Proteome Discoverer. Quantification was based on the reporter ion intensities.

Proteomics data analysis was performed in R through RStudio (RStudio Team, 2020). Proteins that were detected in both tissue and Silk-Ovarioids samples were kept for downstream analysis. Normalized protein contributions were calculated by dividing the protein abundance by the sum of all protein abundances within the sample. One Silk-Ovarioid sample from the cortex was excluded from the analysis due to low peptide concentrations. Subsequently, proteins identified in the proteomics data and presented in the transcriptomics heatmap were selected and scaled protein contributions were used to generate heatmaps. Only samples that were used for both transcriptomics and proteomics analyses were included. Thereafter, a Pearson correlation analysis was performed on data excluding outliers between the normalized RNA counts and normalized protein contributions for tissue and Silk-Ovarioids samples, respectively, using the *cor.test* function in RStudio.

### RNA fluorescent *in situ* hybridization

*In situ* hybridization on formaldehyde-fixed paraffin-embedded (FFPE) Silk-Ovarioids sections (4 μm) was performed using the Multiplex Fluorescent Detection Kit v2 (Advanced Cell Diagnostics, Newark, CA, USA) following the manufacturer’s protocol. In summary, Silk-Ovarioids sections were baked, deparaffinized, and rehydrated before antigen retrieval. Afterward, hybridization with probes for human *AMHR2* (Cat. No. 490241, Advanced Cell Diagnostics), *PDGFRA* (Cat. No. 604481-C2, Advanced Cell Diagnostics), *GJA4* (Cat. No. 856221, Advanced Cell Diagnostics), and *CLDN5* (Cat. No. 517141-C2, Advanced Cell Diagnostics) were performed at 40°C for 2 h. A probe against Ubiquitin C served as a positive control, while a probe detecting the bacterial gene *DabB* was used as a negative control (Advanced Cell Diagnostics). After channel development, sections were incubated with TSA vivid dye 570 and 650 (Tocris, Bristol, UK). Thereafter, DAPI (Advanced Cell Diagnostics) was used to stain nuclei before mounting the samples with prolong Gold anti-fade mounting medium (Life Technologies). Fluorescent-labeled sections were imaged using an inverted widefield Nikon microscope at the Live Cell Imaging (LCI) core facility at Karolinska Institute, Sweden.

### Immunohistochemistry and immunofluorescence of Silk-Ovarioids and tissue sections

For histological evaluation, FFPE Silk-Ovarioids samples were sliced into 4 µm sections and stained with hematoxylin and eosin. The quality of the structure was evaluated through the presence and localization of nuclei in inner and outer parts of the Silk-Ovarioids. Stained sections were imaged with an Olympus IX81 inverted microscope (Carl Zeiss Meditec, Jena, Germany).

Immunofluorescence staining was employed to study the localization of selected markers on FFPE sections of Silk-Ovarioids and tissue samples. Antigen retrieval was performed on deparaffinized and rehydrated 4 µm sections immersed in Tris/EDTA solution (Sigma Aldrich). Blocking solution was composed of 5% w/v bovine serum albumin (Sigma Aldrich), 20% normal donkey serum (Life Technologies), and Tris-buffered saline solution. Sections were blocked for 1.5 h at RT to reduce unspecific binding. Subsequently, primary antibodies against ZO-1, Ki67, γ-H2A.X, cleaved caspase 3, PDGFRα, AMHR2, connexin 37 (Cx37), CLDN5, MCAM, GPIHBP1, collagen 1 α1 (Col1α1), laminin α1 (Lamα1), MMP2, PDGFRβ, BMP2, TGFBR2, and PDGFα were added to the sections for incubation at 4°C overnight. Isotype controls were included as negative controls. Afterward, secondary antibodies incubation was performed at RT for 2 h in the dark. Subsequently, sections were counterstained with DAPI (Thermo Fisher Scientific), mounted with prolong Gold anti-fade mounting medium (Life Technologies), and imaged using an inverted widefield Nikon microscope using 20×/0.75 air objective with 1.5× lens at the LCI core facility. Images were assembled using OMERO.figure and adjusted for brightness and contrast to better visualize the signal. All images were presented on the same scale.

### Terminal deoxynucleotidyl transferase-mediated dUTP nick-end labeling assay

DNA damage in Silk-Ovarioids was assessed using a terminal deoxynucleotidyl transferase-mediated dUTP nick-end labeling (TUNEL) assay (Fluorescence, 594 nm, Cell Signaling Technology, Danvers, MA, USA), according to the manufacturer’s instructions. In brief, sections were deparaffinized, rehydrated, and proceeded to antigen retrieval in citrate solution (Sigma Aldrich). After the TUNEL reaction equilibration, a positive control was prepared by treating the sections with DNase I (>3000 IU/ml, Roche Diagnostics) for 10 min at RT. Sections used as negative controls were incubated with reaction buffer only. Subsequently, TUNEL reaction mix was added to the sections, followed by 2 h incubation (37°C).

### Luminex assays

A bead-based multiplex immunoassay system (Multiplex kit Cytokine & Chemokine 34-Plex Human ProcartaPlex™ Panel 1A, Thermo Fisher, Vienna, Austria) was applied to measure the secretion of cytokines and chemokines by the Silk-Ovarioids samples into the spent culture media after 42 days of culture. In brief, cell debris was removed by centrifuging the supernatants before the assay. The supernatants (50 μl) were then mixed with magnetic beads, as obtained by washing 200 μl of bead mix in a hand-held magnetic plate washer and incubated in the dark at RT for 90 min with continuous shaking. After washing, the beads were incubated sequentially with the detection antibody mix and 50 μl of streptavidin–phycoerythrin solution in the dark at RT (30 min). Thereafter, the washed beads were resuspended, incubated in reading buffer (120 μl) for 5 min with continuous shaking before reading signals. Luminex FLEXMAP 3D instruments coupled with xPONENT 4.3 (DiaSorin, Saluggia, Italy) were used for recording the median fluorescence intensity (MFI). We then converted the MFI and presented the results in pg/ml, which was calculated based on a five-parameter logistic curve.

### Steroid hormones detection and measurement

Steroid hormones were measured in spent culture media after 42 days of culture of Silk-Ovarioids samples using high-performance liquid chromatography coupled with tandem mass spectrometry through an EVOQ Elite Triple Quadrupole mass spectrometer (Bruker, Bremen, Germany) and an Ultimate 3000 UPLC system with a DGP-3600RS dual-gradient pump (Draskau *et al.*, 2019). The limit of quantification (LOQ) for each of the measured hormones is reported. The limit of detection and LOQ were estimated as the concentrations corresponding to 3 and 10 times signal-to-noise ratio, respectively. Data analysis was performed using the software MS Workstation v. 8.2.1.

## Results

### Study setup

**Figure 1.**
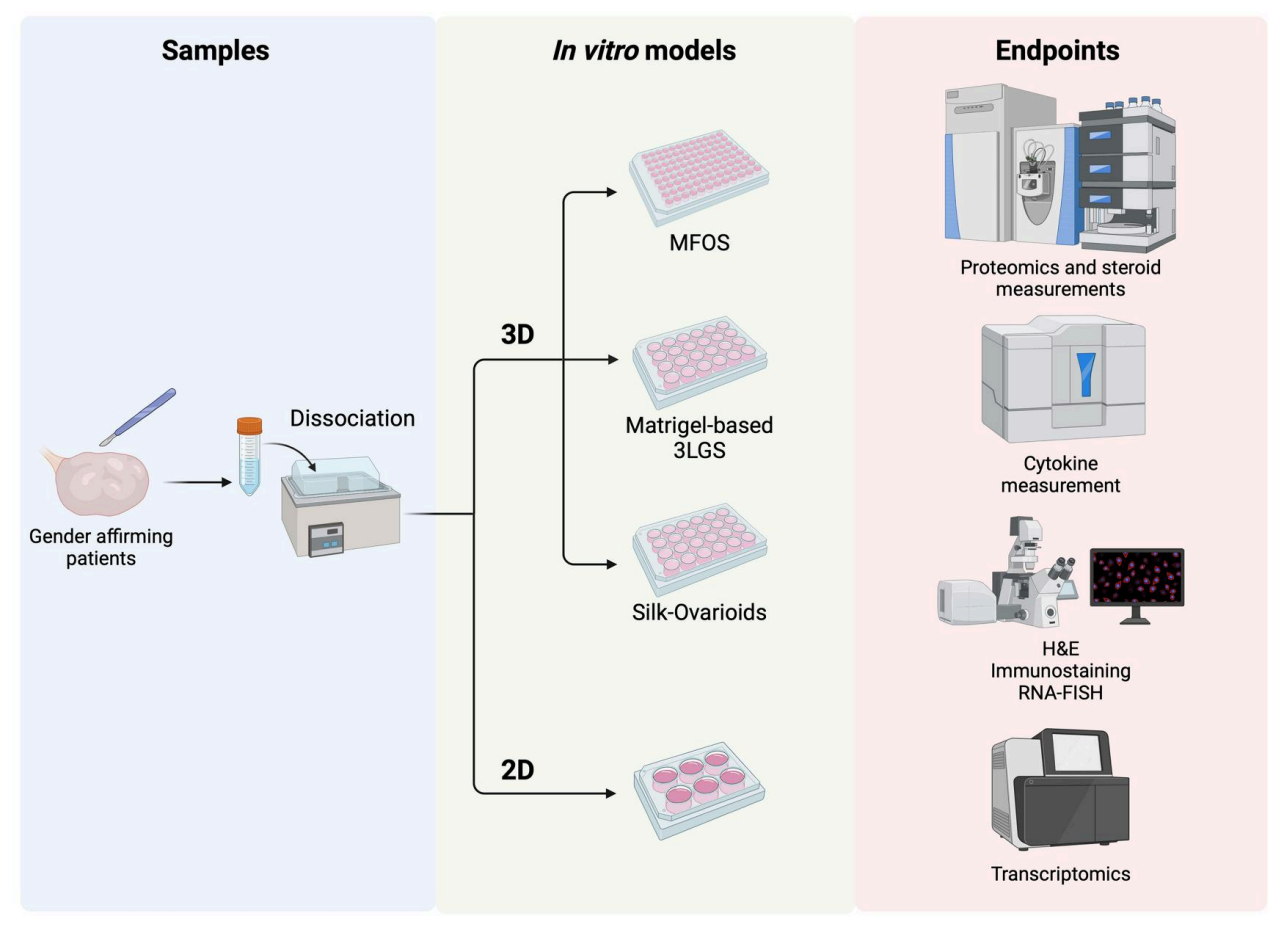
**Overview of experimental setup**. Experimental setup of 2D and 3D *in vitro* models of ovarian primary cells, and endpoints of the study. MFOS: matrix-free ovarian spheroids; 3LGS: three-layer gradient system; H&E: hematoxylin and eosin staining; RNA-FISH: RNA fluorescence *in situ* hybridization. Created with BioRender. Di Nisio, V. (2025) https://BioRender.com/h87o082

In this study, we compared three different approaches to establish a 3D model using adult ovarian primary somatic cells that constitute the natural follicle growth environment in humans: (i) MFOS, (ii) a Matrigel-based 3LGS in hanging drops, and (iii) Silk-Ovarioids. Ovarian tissue samples from five different patients undergoing gender-affirming surgery were dissociated and used for the 3D *in vitro* cultures (Fig. 1). First, we compared the survival of cells in the 3D cultures with those from the same patients in the 2D monolayer cultures. Where stable structures were formed, the quality of the structures was assessed in terms of cell survival/death and morphological and molecular assays (Fig. 1).

The most promising model, the Silk-Ovarioids, was further compared to the human ovarian cortex and medulla via transcriptomics and proteomics analyses, followed by validation of the bioinformatic analysis through immunostaining of the selected markers (Fig. 1). Finally, the functionality of the models was tested by analyzing the secretion of cytokines and steroids (Fig. 1).

### Approaches to grow adult ovarian somatic cells in 3D

**Figure 2.**
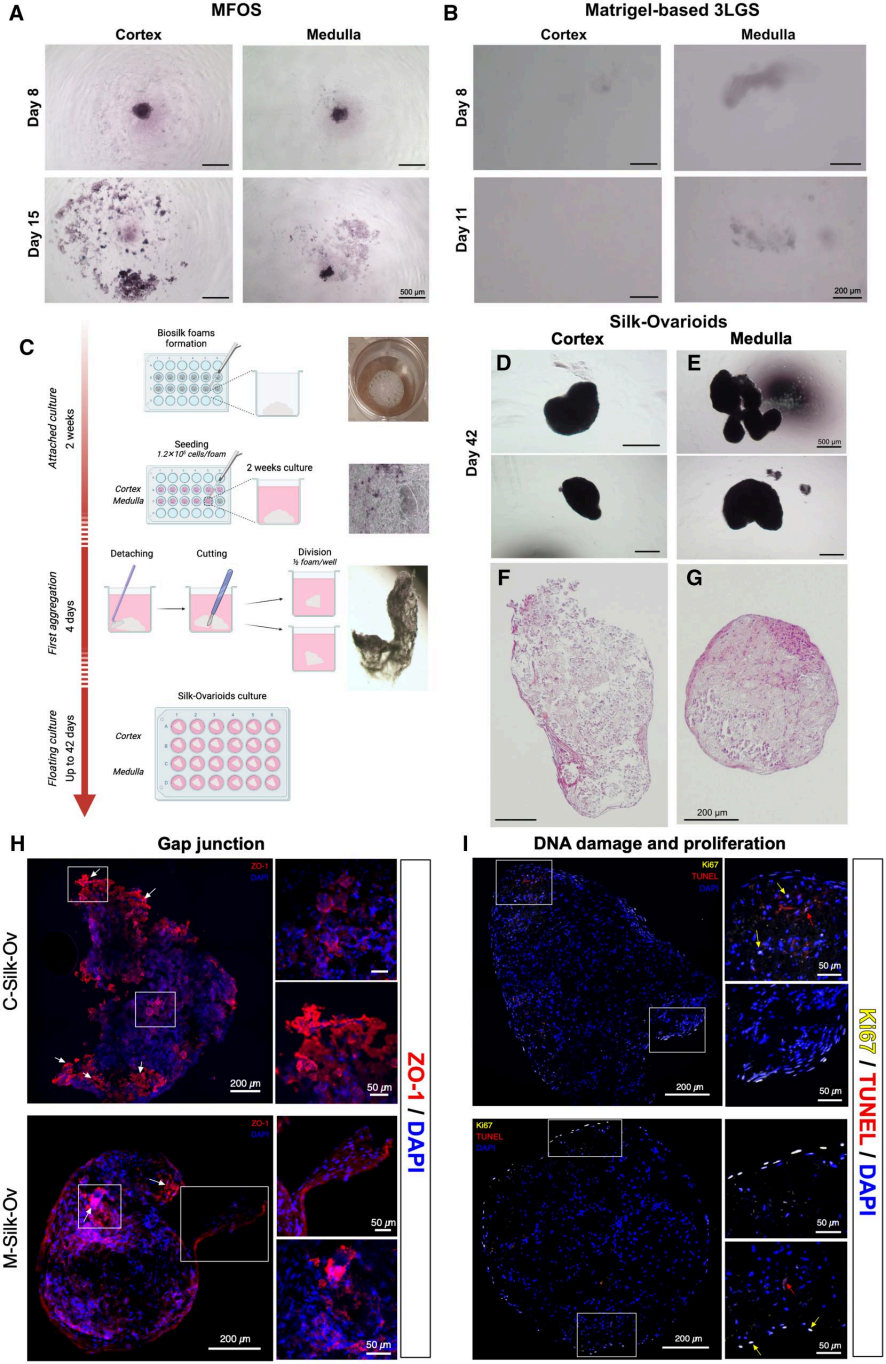
***In vitro* culture of ovarian somatic primary cells**. (**A**) Representative images of MFOS from cortex and medulla in culture. Scale bar represents 500 µm. (**B**) Representative images of Matrigel-based 3LGS in culture. Scale bar represents 200 µm. (**C**) Schematic representation of Silk-Ovarioids formation and culture with the corresponding macroscopic images and timeline (Created with BioRender. Di Nisio, V. (2025) https://BioRender.com/n08v762). Macroscopic morphology of (**D**) C-Silk-Ov and (**E**) M-Silk-Ov. Scale bar represents 500 µm. H&E-stained section of (**F**) C-Silk-Ov and (**G**) M-Silk-Ov. Scale bar indicates 200 µm. Representative images of immunofluorescent staining of gap junction marker ZO-1 (**H**), proliferation marker Ki67 (yellow) and DNA damage marker (TUNEL staining, red) (**I**) in C-Silk-Ov and M-Silk-Ov samples. Arrows of different colors indicate Biosilk background (white), Ki67 (yellow), and the TUNEL (red) fluorescence. Scale bar in the large image represents 200 µm while the scale bar in inserts indicates 50 µm. All images were brightness- and contrast-adjusted for enhanced signal visualization. 3LGS, 3-layer gradient system; C-Silk-Ov, Cortex-derived Silk-Ovarioids; MFOS, matrix-free ovarian spheroids; M-Silk-Ov, Medulla-derived Silk-Ovarioids; TUNEL, terminal deoxynucleotidyl transferase dUTP nick end labeling; ZO-1, zona occludens-1; H&E: hematoxylin and eosin staining.

Adult ovarian somatic cells from the cortex and medulla were cultured in three different models. We first assessed the feasibility of spheroid formation in matrix-free conditions by seeding cells at different densities (3 × 10^4^, 6 × 10^4^, 1.2 × 10^5^ cells/well). MFOS of varying sizes (100–250 µm) were obtained from low and medium cell densities without the support of any matrix in low attachment plates after 8 days of culture; however, these structures spontaneously disintegrated after 2 weeks (Fig. 2A). Concurrently, we applied the Matrigel-based 3LGS culture, previously used to successfully generate rat and human testicular organoids (Alves-Lopes *et al.*, 2017, 2018; Oliver *et al.*, 2021), to ovarian primary cells. Small medulla-derived aggregates (100–200 µm) could be cultured for up to 11 days before the structures began to disintegrate, whereas, surprisingly, no spheroids were formed using cells isolated from cortical tissue (Fig. 2B).

Lastly, ovarian primary cells were seeded on Biosilk scaffolds (Fig. 2C). The seeded scaffolds were left untouched for 2 weeks, remaining attached to the bottom of the well, and were then detached and lifted to promote floating cell-driven aggregation. After 4 days of floating culture, the seeded silk foams started to compact, after which they could be kept in culture for up to 42 days, and harvested for further analyses (Fig. 2D and E). This process was performed for each patient-specific Silk-Ovarioid culture, considered as a separate individual batch (n = 24 Silk-Ovarioids per batch) (Supplementary Table S1). Well-defined structures (400–1000 µm) could be observed using an inverted phase contrast microscope (Fig. 2D and E) and by H&E staining, which confirmed the presence of intact cells throughout the whole structure (Fig. 2F and G). A negative control of cell-free Silk aggregation was included in the first experiment. The cell-free Silk remained as a filamentous scaffold even after 2 weeks of floating culture. A total of five batches of culture using tissues from five patients were carried out with nearly 100% success (Supplementary Table S1).

To determine the quality of the Silk-Ovarioids, we evaluated the *de novo* formation of gap junctions (through the expression of zona occludens-1, ZO-1; Fig. 2H) and markers of DNA fragmentation/proliferation (TUNEL, and Ki67; Fig. 2I). Overall, Silk-Ovarioids showed the presence of gap junctions, a low percentage of apoptotic cells, as also confirmed by the absence of the DNA damage marker, γ-H2A.X, and the cleaved form of caspase 3 (Supplementary Fig. S1), and a low percentage of proliferative cells that are mainly present on the surface of the structure (Fig. 2H and I). Negative controls for immunofluorescence staining and positive control for TUNEL detection are reported in Supplementary Fig. S2.

In summary, the MFOS and Matrigel-based 3LGS culture systems could not support human ovarian somatic cell growth *in vitro*. In contrast, the cells readily grew in Silk-Ovarioids, forming large aggregates that could be maintained for over a month in culture (Supplementary Table S1).

### Silk-Ovarioids harbor all main ovarian cell populations

**Figure 3.**
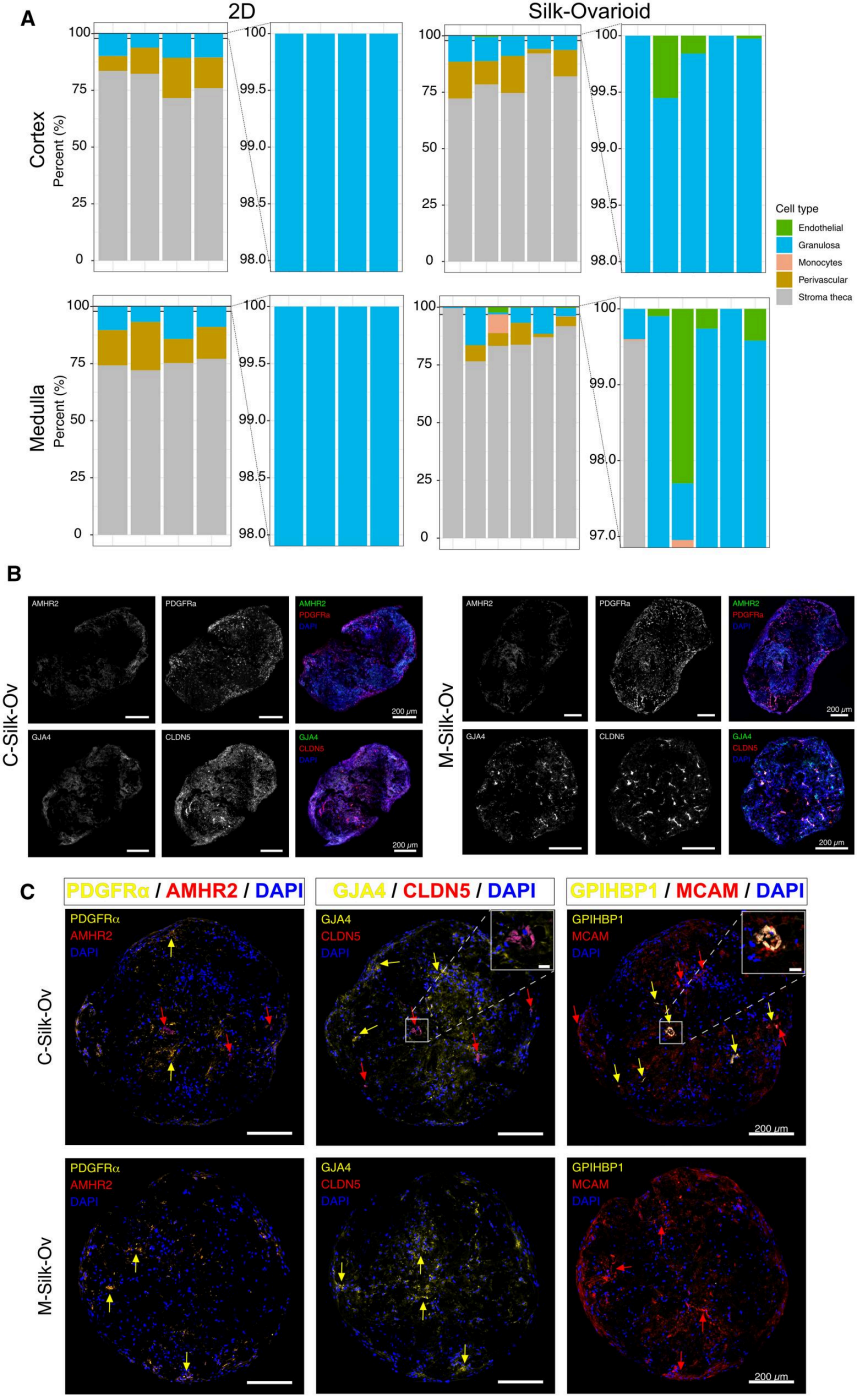
**RNA and protein expression of cell-type markers in Silk-Ovarioids**. (**A**) Composition in percentage of cell types in 2D and Silk-Ovarioids culture systems predicted by dampened weighted least square (DWLS) deconvolution, using a previously published integrated ovarian scRNA-seq data (Wagner *et al.*, 2020) applied to our transcriptomic dataset. Different colors represent different cell types. The magnified stack barplots on the right show the small percentages of endothelial cells in the Silk-Ovarioids. (**B**) RNA fluorescence *in situ* hybridization of stromal (*PDGFRA*), granulosa (*AMHR2*), endothelial (*CLDN5*), and perivascular (*GJA4*) cell markers in C-Silk-Ov and M-Silk-Ov samples (n = 5 each). Scale bar represents 200 µm. (**C**) Protein expression of selected markers in C-Silk-Ov and M-Silk-Ov in consecutive sections. Additional endothelial (GPIHBP1) and perivascular (MCAM) markers were used to confirm the localization of these two cell types. Red and yellow arrows indicate specific marker expression in the C- and M-Silk-Ov. Scale bar in the large image represents 200 µm while the scale bar in inserts indicates 20 µm. All images were brightness- and contrast-adjusted for enhanced signal visualization. AMHR2, anti-Müllerian hormone receptor type 2; CLDN5, claudin 5; C-Silk-Ov, Cortex-derived Silk-Ovarioids; GJA4, Gap junction α 4 (a.k.a. protein connexin 37, Cx37); GPIHBP1, glycosylphosphatidylinositol-anchored high-density lipoprotein-binding protein 1; MCAM, melanoma cell adhesion molecule; M-Silk-Ov, Medulla-derived Silk-Ovarioids; PDGFR-A or -α, platelet-derived growth factor receptor α.

To characterize the cellular composition of Silk-Ovarioids, we deconvoluted the RNA-seq data using previously published scRNA-seq data from ovarian tissue (Wagner *et al.*, 2020). This analysis inferred the cell-type composition in both 2D and Silk-Ovarioid samples. Overall, stromal cells were the predominant cell population irrespective of the culture system adopted. Additionally, the two main cell types identified through transcriptomic prediction were perivascular and granulosa cells (Fig. 3A). Interestingly, we only observed the endothelial cluster in the Silk-Ovarioid samples, but not in the 2D cultures (Fig. 3A). To further validate these findings on Silk-Ovarioids, we analyzed the expression of six markers that are highly specific to different cell types: PDGFRA (stromal cells), AMHR2 (granulosa cells), GPIHBP1, and CLDN5 (endothelial cells), and MCAM and GJA4 (perivascular cells). As a positive control, we used a cross section of an ovary for the identification of these markers in the corresponding structures in the tissue (Supplementary Fig. S3). Their expressions were analyzed in cortex- (n = 5) and medulla-derived (n = 5) Silk-Ovarioids through RNA fluorescent *in situ* hybridization (RNA-FISH) and/or immunofluorescence staining (Fig. 3B and C).

As shown in Fig. 3B and C, all the selected markers were detected in the Silk-Ovarioid samples, both at the mRNA and protein levels. All Silk-Ovarioids showed a high mRNA expression of *PDGFRA* (Fig. 3B), although its protein levels were less pronounced (Fig. 3C). Signals for AMHR2 were sparce on both mRNA and protein levels, indicating that granulosa cells are present in very low numbers. Markers for endothelial cells (GPIHBP1 and CLDN5) and perivascular cells (MCAM and GJA4, also known as Connexin 37, Cx37) were found in the inner and outer part of Silk-Ovarioids and were localized near each other (Fig. 3B and C). Finally, no oocytes were detected in the heterogeneous population of somatic cells forming the Silk-Ovarioids, likely due to the stringent straining used prior to seeding.

After 6 weeks of culture, the development of tubular structures in the core of Silk-Ovarioids derived from the cortex was observed. These structures were positive for the endothelial cell markers CLDN5 and GPIHBP1, suggestive of blood vessel-like formation (Fig. 3C, upper panel inserts).

### Silk-Ovarioids culture upregulates angiogenesis-related genes

**Figure 4.**
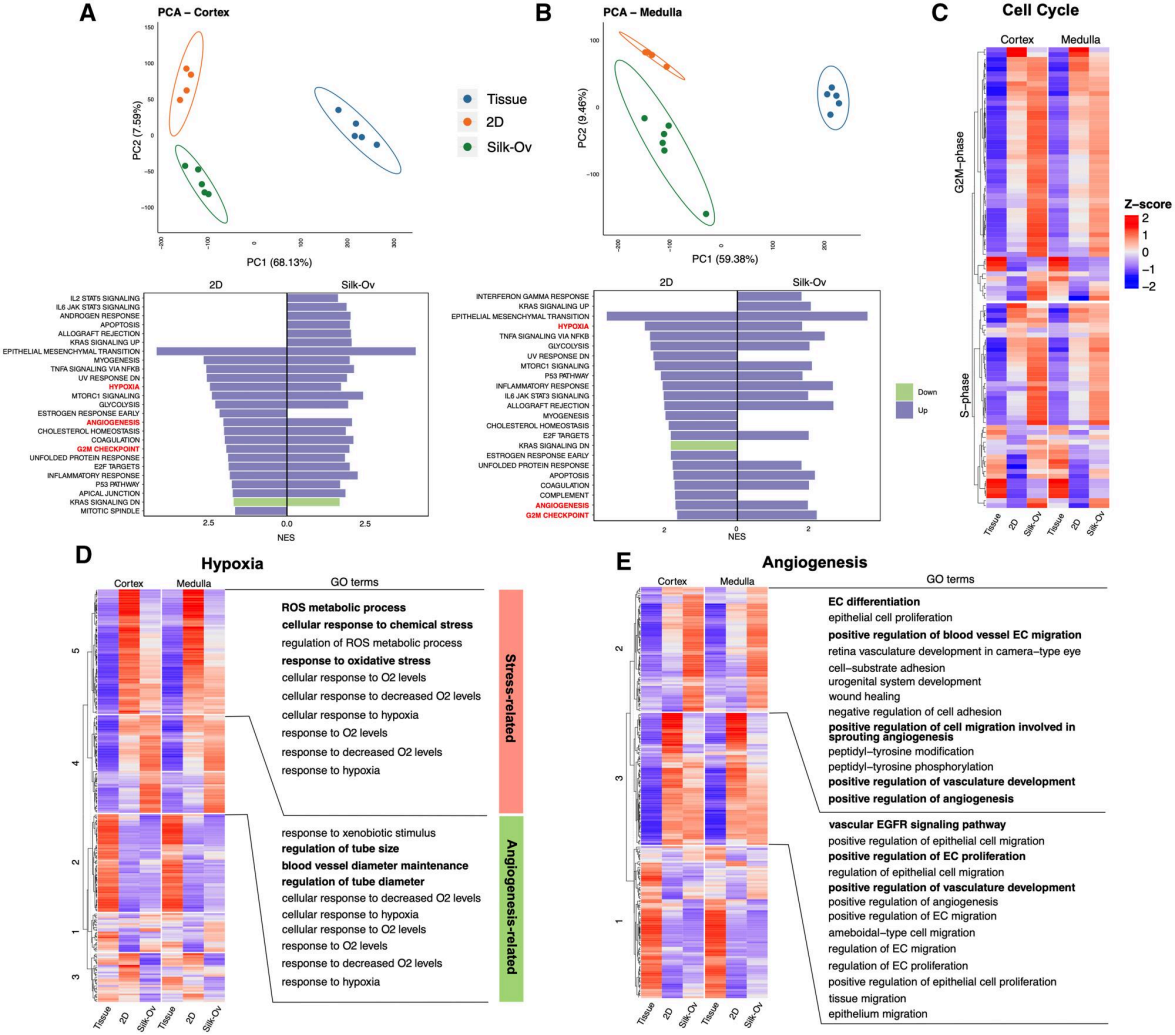
**Transcriptomics profiling of tissue, 2D cell culture, and Silk-Ovarioids (Silk-Ov) samples**. (**A**) PCA of tissue (n = 5), 2D cell culture (n = 4) and Silk-Ov (n = 5 for cortex; n = 6 for medulla) samples originating from ovarian cortex primary cells from five patients. Enriched hallmark gene sets using DEGs from the 2D versus tissue and Silk-Ov versus tissue comparisons. One Silk-Ov sample from cortex was excluded due to low library size. (**B**) PCA of tissue (n = 5), 2D (n = 4), and Silk-Ov (n = 6) samples originated from ovarian medulla primary cells from five patients. Enriched hallmark gene sets using DEGs from the 2D versus tissue and Silk-Ov versus tissue comparisons. Normalized enrichment score (NES) is presented in the *x*-axis. Purple represents upregulation while light green represents downregulation. (**C**) Heatmap of average *z*-scores of G2M and S phase marker genes in cortex and medulla samples. (**D**) Heatmap of average *z*-scores of genes related to hypoxia in cortex and medulla samples (left). Genes are clustered using k-means clustering. Top 10 GOs enriched in cluster 5 and cluster 4 are shown (right). Stress-related cluster is shown in pink while angiogenesis-related cluster is presented in green. (**E**) Heatmap of average *z*-score of genes related to angiogenesis in cortex and medulla samples (left). Genes are clustered using k-means clustering. Top GOs enriched in cluster 2 and cluster 3 are shown (right). Counts are normalized using DESeq2 normalization and scaled to obtain mean equals 0 and SD equals 1. The final gene expression is represented using *Z*-scores. EC, endothelial cells; GO, gene ontology; NES, normalized enrichment score; ROS, reactive oxygen species; DEGs, differentially expressed genes.

To further characterize the Silk-Ovarioid model, we performed RNA sequencing (n = 5 for cortex; n = 6 for medulla) and compared them to ovarian tissue (n = 5) and 2D cell cultures (n = 4) from the same patient and cultured for the same period of time alongside Silk-Ovarioids. Transcriptomic profiling of freshly collected ovarian tissues, 2D cultured cells, and Silk-Ovarioids showed a clear separation between fresh tissues and cultured samples in both cortex and medulla, explaining 68.1% and 59.4% of the difference in PC1 in principal component analysis (PCA), respectively (Fig. 4A and B). The ovary is a complex organ composed of numerous cell types, which posed a challenge for data normalization between freshly collected samples and the *in vitro* system. The latter contains only the selected cells that survived the culture. Therefore, we applied strict criteria (i.e. FDR<0.05, absolute log_2_FC>2, and average expression>100) to define DEGs and to remove false positive results. As expected, the largest number of DEGs was identified when tissue was compared to cultured samples (Cortex: tissue vs 2D culture, 2808 DEGs; tissue versus Silk-Ovarioids, 2533 DEGs. Medulla: tissue versus 2D culture, 2494 DEGs; tissue versus Silk-Ovarioids, 2246 DEGs) (Supplementary Fig. S4A; Supplementary Table S2). The heatmaps of the top 500 most variable genes confirmed a clear separation among the three types of samples (Supplementary Fig. S4B). On the other hand, comparing Silk-Ovarioids to 2D samples revealed relatively subtle effects in both cortex and medulla, where 553 and 618 DEGs were found with the same cutoff criteria, respectively (Supplementary Fig. S4A).

To assess the impact of different culture systems on the transcriptome compared to the fresh tissue controls, we performed GSEA against hallmark gene sets using significant DEGs ranked by log_2_FC. GSEA results revealed a similar change induced by culture in both 2D and Silk-Ovarioid contexts, i.e. the upregulation of genes involved in the cell cycle G2M checkpoint, angiogenesis, hypoxia, and TNFα signaling via NF-κB pathways in both cortex- and medulla-derived samples (Fig. 4A and B). However, we also observed changes specific to Silk-Ovarioid samples such as IL6–JAK–STAT3 signaling in the cortex and interferon-γ response in medulla-derived Silk-Ovarioids (Fig. 4A and B).

Focusing on the commonly affected pathways in 2D and Silk-Ovarioids samples, i.e. hypoxia and angiogenesis, we plotted the average expression of associated genes collected from the GO database. For changes related to the cell cycle, we extracted the S- and G2M-phase genes used for cell cycle scoring in the Seurat package. Silk-Ovarioids samples appeared to have a high proliferative profile compared to that of 2D cultures and tissue, as indicated by a higher expression of G2M and S phase markers (Fig. 4C). Additionally, samples cultured in 3D conditions displayed a relatively lower hypoxia response compared to that of 2D cultures, as shown in the gene set enrichment score plot (Fig. 4A and B).

Using a classical machine-learning method, k-means clustering, we grouped hypoxia-related genes into five main clusters (Fig. 4D). Two identified patterns were culture-specific, where the hypoxia-5 cluster genes were highly expressed in 2D samples, and the hypoxia-4 cluster was upregulated in Silk-Ovarioids samples (Fig. 4D). To further investigate the biological processes associated with these genes, we performed a GO over-representation analysis. Genes in the hypoxia-5 cluster showed significant enrichment for stress-related GOs in 2D cultures, such as reactive oxygen species metabolic process and responses to oxidative stress (Fig. 4D; Supplementary Table S3). On the other hand, genes in the hypoxia-4 cluster were enriched in angiogenesis-related GOs in the Silk-Ovarioids, i.e. regulation of endothelial tube size, diameter, and blood vessel diameter maintenance (Fig. 4D; Supplementary Table S3).

We next characterized changes related to angiogenesis signaling. Genes that were highly expressed in Silk-Ovarioids samples were associated with endothelial cell differentiation, regulation of blood vessel endothelial cell migration, and sprouting angiogenesis (Fig. 4E; Supplementary Table S4). In the 2D-specific cluster, genes were related to vascular EGFR signaling and regulation of endothelial cells (Fig. 4E; Supplementary Table S4). These observations were further confirmed by GO enrichment analysis on significant DEGs, ranked by log_2_FC, identified in the Silk-Ovarioid and 2D comparisons. In line with these results, GOs related to angiogenesis (i.e. vasculature and blood vessel development, sprouting angiogenesis) were significantly upregulated in cortex-derived Silk-Ovarioids samples. Similarly, endothelium development and sprouting angiogenesis were significantly upregulated in Silk-Ovarioid cultures compared to 2D samples derived from the medulla (Supplementary Table S4).

Collectively, these findings suggest that both cortex and medulla-derived Silk-Ovarioids have a higher cellular proliferation potential at the transcriptomic level compared to their respective 2D systems. Moreover, culture conditions induced hypoxia-associated markers in both 2D and 3D models. The main difference is the upregulation of stress-related hypoxia markers in 2D cultures and the predominant increase of pro-angiogenic-related markers in Silk-Ovarioids.

### Silk-Ovarioids culture enables *de novo*-angiogenesis through formation of hypoxic environment

We further profiled single Silk-Ovarioids derived from both the cortex and medulla at the protein level. In total, three Silk-Ovarioids and the corresponding freshly fixed tissues from both the cortex and medulla of three different patients were subjected to individual proteomic analysis using liquid chromatography–tandem mass spectrometry (LC–MS/MS). In both tissue and Silk-Ovarioids, we detected peptides involved in biological processes mainly related to transport and signal transduction, RNA and protein metabolism, cell–cell communication, and cell organization, adhesion, and proliferation.

**Figure 5.**
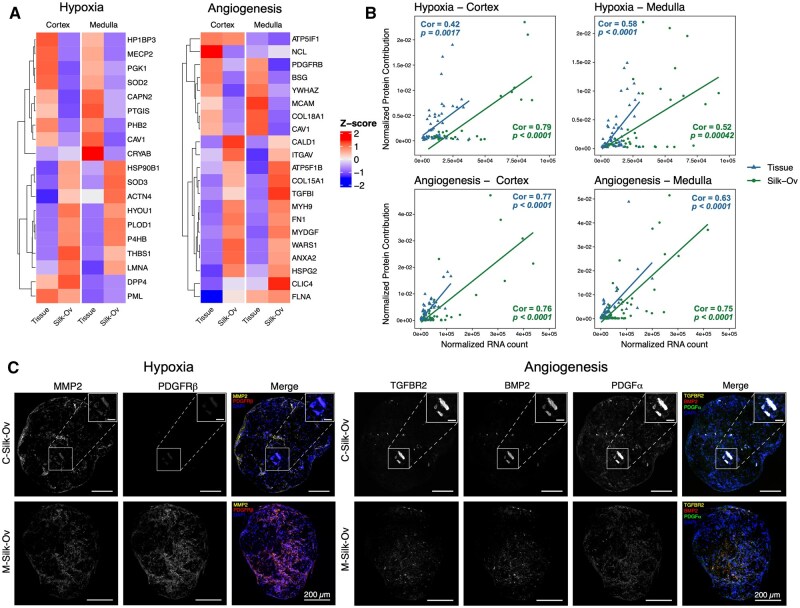
**Proteomics and immunostaining of hypoxia- and angiogenesis-related markers in Silk-Ovarioids**. (**A**) Average normalized protein contribution of hypoxia- and angiogenesis-related markers in tissue and C- and M-Silk-Ov samples (n = 2 for cortex and n = 3 for medulla). One Silk-Ovarioids (Silk-Ov) sample from the cortex was excluded due to low peptide concentrations. Expression was normalized to obtain mean equals to 0 and SD equals to 1. (**B**) Pearson correlation between detected normalized hypoxia and angiogenesis proteins and normalized RNA counts in RNA-seq data (19 markers). RNA counts were normalized using DESeq2 normalization. One Silk-Ov sample from the cortex was excluded due to low library size. Normalized protein contribution indicates the relative expression of a protein to the total amount of peptides detected. One Silk-Ov sample from the cortex was excluded due to low peptide concentrations. Tissue and C- and M-Silk-Ov samples were analyzed separately. The regression lines are plotted using linear model fitting. The correlation coefficient value is shown as Cor and *P*-value is given in the plots. Data from tissue are shown as blue triangles while data from Silk-Ov are presented as green round dots. (**C**) Representative images of immunofluorescence staining for hypoxia-related markers (MMP2, PDGFRβ) and angiogenesis-related markers (TGFBR2, BMP2, PDGFα) in sequential sections of C- and M-Silk-Ov (n = 5 each). Inserts show higher magnification images of the vessel-like structures in the core of Silk-Ov. Scale bar in the large image represents 200 µm while scale bar in inserts indicates 50 µm. BMP2, Bone morphogenetic protein 2; C-Silk-Ov, Cortex-derived Silk-Ovarioids; MMP2, Matrix metallopeptidase 2; M-Silk-Ov, Medulla-derived Silk-Ovarioids; PDGFα, Platelet-derived growth factor α; PDGFRβ, Platelet-derived growth factor receptor β; TGFBR2, Transforming growth factor-β receptor type 2.

Due to the relatively low number of peptides detected per Silk-Ovarioid, we analyzed the normalized protein contribution as their relative expression to the total amount of peptides detected for each sample. The heatmap of proteins involved in hypoxia and angiogenesis signaling revealed two distinct clusters, indicating an upregulation of pro-angiogenic hypoxic environment and *de novo* angiogenesis markers (Fig. 5A). Most of the proteins that had a high contribution in Silk-Ovarioid samples were previously identified in the transcriptomics analysis as angiogenesis-related genes (Fig. 5A). Next, we compared the protein and RNA levels of the hypoxia- and angiogenesis-related genes through Pearson correlations. We recorded moderate to strong correlations between the normalized protein contribution and normalized RNA counts both in cortex and medulla (Fig. 5B).

To further validate the protein–RNA correlation, we selected highly expressed markers of hypoxia (i.e. MMP2 and PDGFRβ) and angiogenesis (i.e. TGFBR2, BMP2, and PDGFα) from the differential expression analysis and investigated their protein localization via immunofluorescent staining. The results confirmed the presence of hypoxic and angiogenic proteins, and their localization highlighted a functional asset of the expressed markers. In fact, the pro-angiogenic environment was surrounded and initiated by hypoxic pockets that disappeared when endothelial cells were reorganized into vessel-like structures (Fig. 5C). For instance, Fig. 5 shows representative images of a cortex-derived Silk-Ovarioid already containing a vessel (Fig. 5C, upper panel) and a medulla-derived Silk-Ovarioid in which the sprouting of new vessels was still not evident (Fig. 5C, lower panel). As hypothesized, when the formation of the vessel commences, the hypoxia-related markers are downregulated in the inner part of the cortex-derived Silk-Ovarioid, while the newly created vessel showed colocalization of all three angiogenesis-related markers (Fig. 5C, upper panel). Mirroring this pattern, in the medulla-derived Silk-Ovarioids, where the formation of the vessels was still in progress, the hypoxic markers were mainly identified in the center of the 3D model (Fig. 5C, lower panel). Concomitantly, the angiogenic markers were faintly present and localized in the core of the Silk-Ovarioid, surrounded by the pro-angiogenic hypoxic environment (Fig. 5C, lower panel).

Collectively, these observations suggest that some of the main markers driving hypoxia and angiogenesis were detected in the proteomics analysis, positively correlating with the transcriptomic counterpart in both tissue and Silk-Ovarioids. Moreover, *de novo* angiogenesis could be observed in the center of the Silk-Ovarioid samples, initiated by the formation of a hypoxic environment.

### Silk-Ovarioids enable ECM formation and remodeling during culture

**Figure 6.**
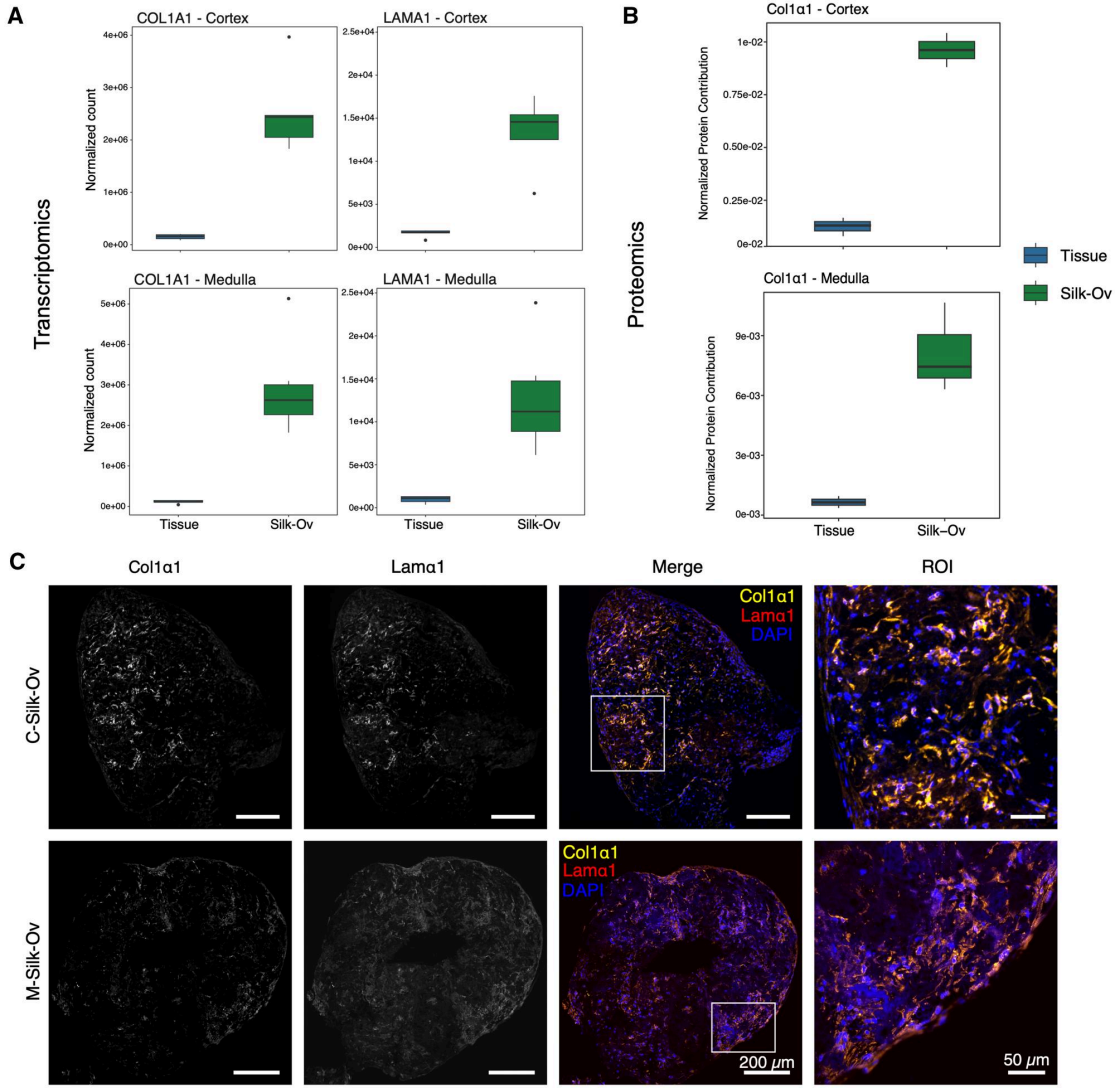
**Expression of ECM markers in tissue and Silk-Ovarioids (Silk-Ov) samples in cortex and medulla**. (**A**) Normalized expression of top-expressed ECM-related markers *COL1A1* and *LAMA1* in tissue (n = 5) and Silk-Ov (n = 5 for cortex; n = 6 for medulla) samples in cortex and medulla as measured by RNA-seq. One Silk-Ov sample from the cortex was excluded due to low library size. RNA counts were normalized using DESeq2 normalization. (**B**) Normalized protein contribution of Col1α1 in proteomics data from tissue and Silk-Ov samples (n = 3 each). Normalized protein contribution indicates the relative expression of a protein to the total amount of peptides detected. One cortex Silk-Ov sample was excluded due to low peptide concentrations. Dots represent biological replicates in each group. (**C**) Representative images of Col1α1 and Lamα1 immunofluorescence staining in C-Silk-Ov and M-Silk-Ov (n = 5 each). The ROIs highlight the presence of ECM markers after cell secretion. Scale bar for large images represents 200 µm, while in inserts, scale bar indicates 50 µm. Col1α1, Collagen type 1 α1; C-Silk-Ov, Cortex-derived Silk-Ovarioids; ECM, extracellular matrix; Lamα1, Laminin subunit α1; M-Silk-Ov, Medulla-derived Silk-Ovarioids; ROI, region of interest.

When comparing the transcriptomic profile of tissue and Silk-Ovarioids, the differential expression analysis and GO enrichment analysis identified a set of highly modulated targets related to ECM organization (Supplementary Table S5). Among these, we selected the most highly expressed genes, *COL1A1* and *LAMA1*, for further validation. Interestingly, we recorded a significantly higher expression of these two markers in RNA-seq data from both cortex- and medulla-derived Silk-Ovarioids compared to tissue, while at the protein level, only collagen type 1 α1 (Col1α1) expression was detected, showing a trend consistent with the transcriptomic data (Fig. 6A and B). The presence of *de novo* secretion of these ECM-related proteins was further confirmed by immunofluorescence staining (n = 5 for both cortex- and medulla-derived Silk-Ovarioids from five patients), using antibodies listed in Supplementary Table S6. The colocalization of both Col1α1 and laminin subunit α 1 (Lamα1) was detected in the core and outer part of the Silk-Ovarioids samples from both cortex and medulla (Fig. 6C). On the other hand, the 2D monolayer cells showed low or no expression of these ECM markers, in comparison with both tissues and Silk-Ovarioids (Supplementary Fig. S5).

### Silk-Ovarioids secrete pro-angiogenic cytokines and steroids

**Figure 7.**
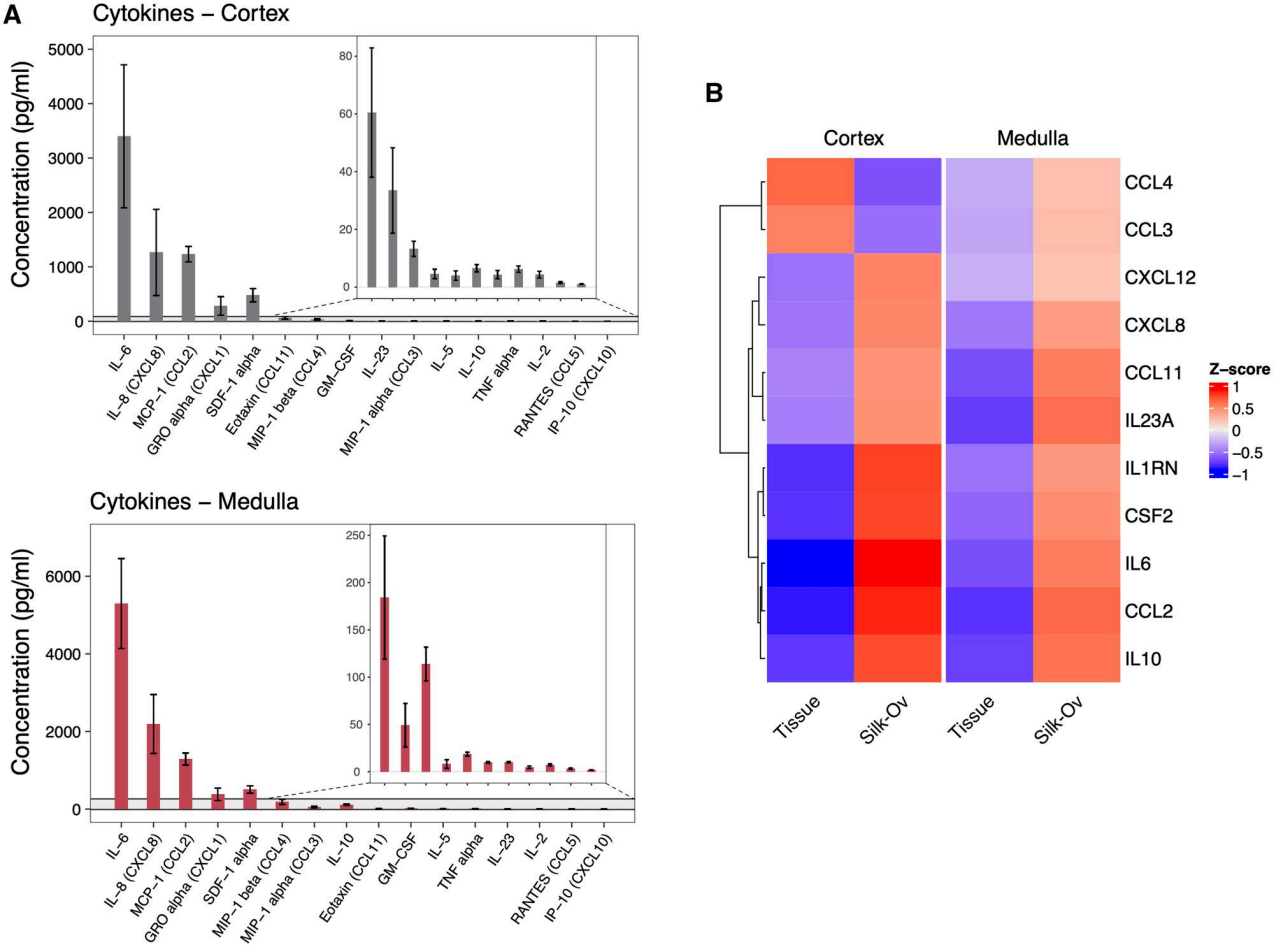
**Expression and secretion of cytokines by Silk-Ovarioids**. (**A**) Concentration of secreted cytokines and chemokines in the culture media collected from cortex and medulla Silk-Ovarioids (n = 4 biological replicates and 3 technical replicates each) at Day 42 of culture. Results are presented as bar plot with the mean±SEM. (**B**) Heatmap of average *z*-score of genes encoding the culture medium-detected cytokines in tissue (n = 5) and Silk-Ov (n = 5 for cortex; n = 6 for medulla) samples in cortex and medulla, based on RNA-seq data. One Silk-Ov sample from the cortex was excluded due to low library size. Counts were normalized using DESeq2 normalization and scaled to obtain mean equals 0 and SD equals 1. Silk-Ov, Silk-Ovarioids.

We conducted functional studies by measuring the cytokines and steroids secreted during culture by the Silk-Ovarioids. To cover a wide spectrum, we used a multiplex panel that included 34 cytokines in the analysis. Similar cytokines were detected in the collected medium after 42 days of culture in both cortex- and medulla-derived Silk-Ovarioids (n = 4), with the most abundant being IL-6, IL-8, MCP-1, CXCL1, and SDF-1 alpha (Fig. 7A). As the cytokines were measured in culture medium, we could not compare the profiles between tissue and Silk-Ovarioids. Therefore, we examined the differences in the corresponding genes between tissue and Silk-Ovarioids samples using the RNA-seq data. Consistent with the secreted cytokines, the selected cytokine-encoding genes were highly expressed in Silk-Ovarioid samples compared to cortex and medulla tissue (Fig. 7B). Interestingly, all the detected cytokines in the medium, particularly the most highly expressed ones (e.g. IL-6, IL-8, and GM-CSF), are shown to be pro-angiogenic, stimulating endothelial cell proliferation and migration. This suggests they may play a role in supporting the formation of new vessels under physiological conditions (Kushner *et al.*, 2010).

To assess the production and secretion of steroids in the culture media, we performed LC–MS/MS on a panel of 15 hormones, including androstenedione, corticosterone, cortisol, dehydroepiandrosterone, deoxycortisol, dihydrotestosterone, epitestosterone, hydroxycortisol, hydroxyprogesterone, pregnenolone, progesterone, testosterone, aldosterone, estrone, and β-estradiol (Supplementary Table S7). In our samples, we identified four main steroids in the spent culture media of cortex- and medulla-derived Silk-Ovarioids (n = 3) after 42 days of culture. Generally, steroids were present at very low levels, often below the LOQ. Pregnenolone and epitestosterone were the most detected steroids above the LOQ, while the detection rates of estrogens (estrone and β-estradiol) were much lower (Supplementary Table S7). Additionally, to provide a general overview of the steroidogenesis-related gene expression, we compared the transcriptomic profiles between Silk-Ovarioids and tissue. The heatmap of well-known steroidogenic enzymes and related regulators showed a downregulation of the typical granulosa markers (i.e. *FOXL2*, *KIT*, *AMH*, *AR*) and oocyte-specific markers (i.e. *GDF9*) in Silk-Ovarioid samples (Supplementary Fig. S6). However, we observed an increased expression of *CYP19A1*, along with members of the nuclear receptor family (e.g. *ESRRA*, *ESRRB*, *NR4A3*), and steroidogenesis-related growth factors and receptor genes (e.g. *GNDF*, *FGF2*, *IGF2R*) (Supplementary Fig. S6). Although Silk-Ovarioids displayed a lower expression profile compared to tissue samples, the presence of *CYP11A1* justifies the low but detectable secretion of pregnenolone in the culture medium. Additionally, the upregulation of genes encoding for E1- and E2-synthesizing enzymes, *AKR1C3* and *CYP19A1*, respectively, was evidenced in Silk-Ovarioids compared to the tissue counterpart.

### Silk-Ovarioids as a potential new scaffold for ovary models

**Figure 8.**
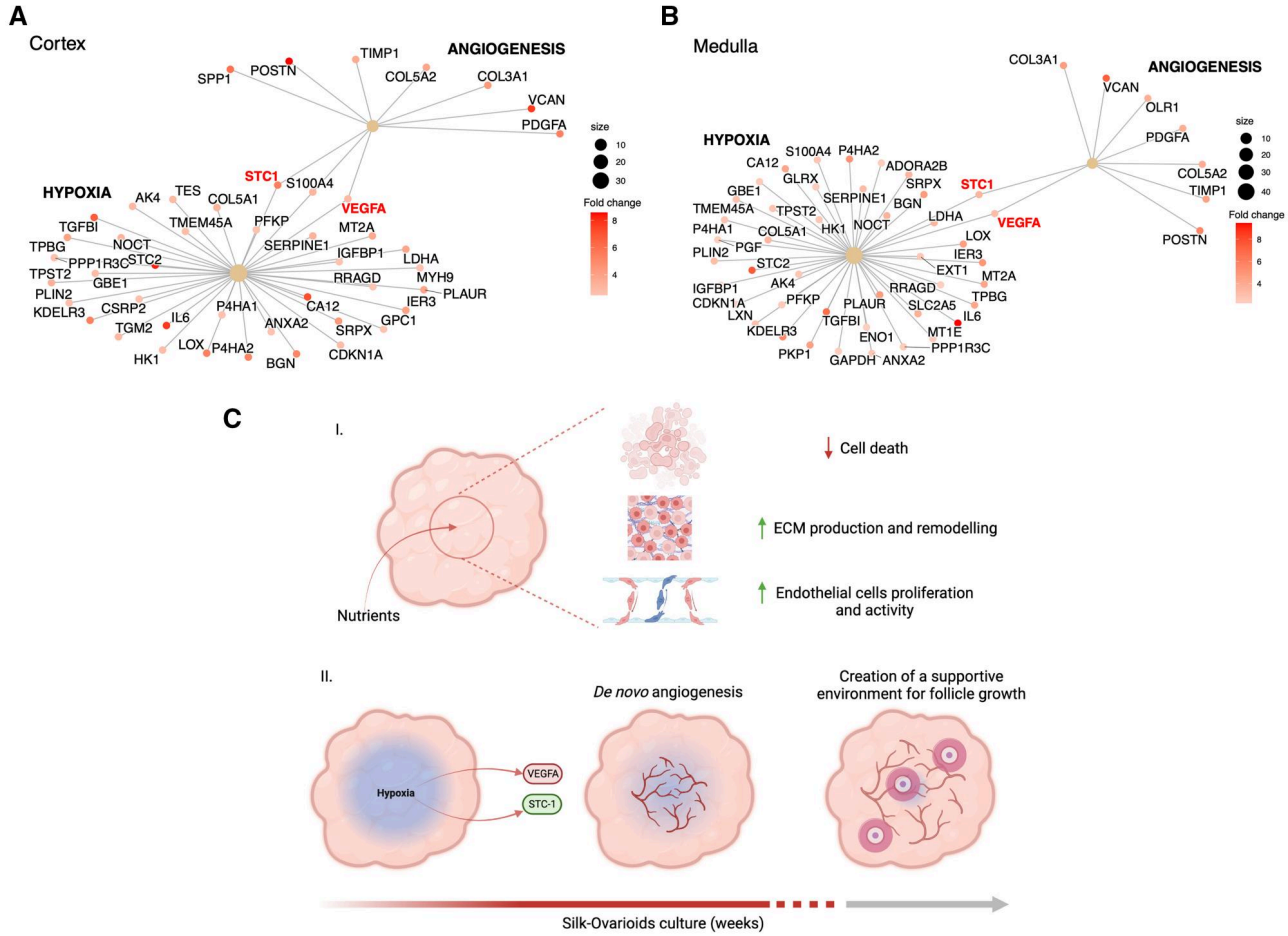
**Gene-concept network and schematic overview of Silk-Ovarioids**. Gene-concept network of hypoxia and angiogenesis signaling using significant DEGs identified in comparison of Silk-Ovarioids and tissue samples from cortex (**A**) and medulla (**B**). The color scale of the gene dots indicates fold change. The size of the central hubs’ dots (hypoxia and angiogenesis) represents the number of DEGs enriched in each gene set. (**C**) Schematic overview of (**I**) main ongoing mechanisms and (**II**) hypoxia-triggered angiogenesis process in Silk-Ovarioid culture (Created with BioRender. Di Nisio, V. (2025) https://BioRender.com/b76r069). ECM, extracellular matrix; STC-1, stanniocalcin 1; VEGFA, vascular endothelial growth factor A; DEGs, differentially expressed genes.

To better visualize the interplay between hypoxia and angiogenesis in our samples, we used the gene-concept network. Specifically, we examined the connections of DEGs identified in the comparison of Silk-Ovarioids versus tissue, focusing on those involved in hypoxia and angiogenesis in both cortex and medulla (Fig. 8A and B). Intriguingly, crosstalk between hypoxia and angiogenesis signaling was discovered in both cortex- and medulla-derived Silk-Ovarioids, mediated by two genes, *STC1* and *VEGFA* (Fig. 8A and B). These factors, namely stanniocalcin-1 and vascular endothelial growth factor A, are not only well-known regulators of angiogenesis but also involved in follicular activation and growth (Bishop *et al.*, 2021; Guzmán *et al.*, 2023).

Altogether, the deep molecular characterization of the Silk-Ovarioids model showcases a promising new *in vitro* system that offers several advantages over other comparable models (Fig. 8C): the silk scaffold allows for the attachment and long-term stable growth of human adult ovarian somatic cells, preserving cellular diversity; it triggers the cells to generate their own natural ECM; it promotes the passage of nutrients thus avoiding the formation of a necrotic core; and it enables the establishment of a pro-angiogenic hypoxic environment promoting proliferation and migration of endothelial cells as well as the formation of vessel-like structures (Fig. 8C-I and II). We hypothesize that the sprouting of these small channels may improve oxygenation in the core of Silk-Ovarioids, acting as a negative feedback mechanism to suppress the formation of a hypoxic environment (Fig. 8C-II). Furthermore, the model showed little variability between samples and patients, which will be important for potential future patient-specific applications. In summary, Silk-Ovarioids can be considered as autonomous structures that, with the proper supplementation of growth and nutrition factors, can likely establish a supportive self-sustaining environment for *in vitro* follicular growth (Fig. 8C-II).

## Discussion

This study introduces a novel, reproducible, and robust silk-based 3D model of primary human somatic cells derived from healthy ovaries. In contrast to previous Matrigel-based approaches for culturing patient-derived ovarian cancer cells (Nanki *et al.*, 2020; Maenhoudt and Vankelecom, 2021; Yang *et al.*, 2021) or testicular and ovarian organoids (Alves-Lopes *et al.*, 2017; Oliver *et al.*, 2021), our Silk-Ovarioids model facilitates the survival and proliferation of the main somatic cell populations of the ovary, along with the autonomous establishment of a hypoxic environment crucial for subsequent neo-angiogenesis.

The Matrigel-based 3LGS method efficiently establishes fetal gonadal organoids, supporting the maintenance of ovarian germ cells isolated from first-trimester gonads (Oliver *et al.*, 2021). However, when applying the matrix-free and 3LGS protocols to adult ovarian samples, the cells appeared incapable of either aggregating or surviving in culture for extended periods. A potential explanation for this discrepancy is the stemness of adult ovary samples. Adult cells can be classified as multipotent or completely differentiated cells, with very low levels of stemness markers (Supplementary Table S2), and limited capacity for multiple cell divisions (Łos *et al.*, 2019). This characteristic, which contrasts with ovarian cancers and fetal tissues, led us to employ the silk-based system, which is xeno-free and potentially compatible with future clinical applications. Although the system has been applied in 3D cultures of human pluripotent stem cells (Fiorenzano *et al.*, 2021; Sozzi *et al.*, 2022) and several primary cell types (Johansson *et al.*, 2019), it has not yet been utilized for ovarian cells. In this study, seeding ovarian primary cells on silk foams enabled their attachment and growth while still attached to the plate. Subsequently, following detachment and division of the seeded foams, all Silk-Ovarioids began to condense and aggregate after 4 days, and were successfully maintained in suspension culture for up to 42 days. The condensation of the foams to create well-defined and compacted structures was solely driven by cell-to-cell interactions, as cell-free silk foams did not reach a compact stage even after 14 days of culture in suspension. This study thus represents the first description of relatively large 3D structures (ranging from 400 to 1000 µm) derived from ovarian primary cells that can be stably cultured for extended periods. We chose 42 days of culture as a preliminary timepoint to assess the molecular and structural processes ongoing in the newly established model, and the analysis showed no signs of cell death or distress. To date, ongoing experiments are testing longer culture times for Silk-Ovarioids growth and maintenance, and two batches have been kept in culture for up to 168 days (nearly half a year) without showing signs of cell death or structure disaggregation (data not shown).

The quality of the newly established Silk-Ovarioids model was evaluated by examining their internal structures and assessing the health status of the cells in terms of DNA fragmentation and proliferation. A primary concern with 3D cultures is the development of a necrotic core due to limited nutrient and oxygen exchange (Huang *et al.*, 2017). In the Silk-Ovarioids, DNA fragmentation was nearly undetectable, indicating the absence of a necrotic core typically observed in structures exceeding 400 µm in diameter (Huang *et al.*, 2017). Additionally, proliferation, as indicated by Ki67 expression, was mainly localized to the outermost thin layer of cells surrounding both cortex- and medulla-derived Silk-Ovarioids. This 3D model establishes a hypoxic environment in the inner core, partially resembling multicellular tumor spheroids (Giverso and Preziosi, 2019). It is well-documented that a necrotic core induces hypoxia in the inner region of multicellular tumor spheroids, inhibiting cell division and prompting the surrounding cells to enter a quiescent state (Giverso and Preziosi, 2019). Thus, the cells inside tumor spheroids remain alive but non-proliferative, while proliferation continues in the outer shell (Giverso and Preziosi, 2019). In Silk-Ovarioids, the formation of a necrotic core is prevented by the silk-based scaffold, as also observed in similar systems such as human brain organoids (Fiorenzano *et al.*, 2021). However, the silk-based structure allows the formation of hypoxic pockets, which in turn stimulate the initiation of pro-angiogenic mechanisms. Further support was found in the active secretion of pro-angiogenic cytokines into the culture media.

In general, the formation of new gap junctions during organoid culture, concomitant with autonomous ECM secretion, indicates effective cell communication and proper remodeling of the scaffold. We confirmed tight cell adhesion in both the inner and outer regions of the model across five batches of Silk-Ovarioids, substantiated by the presence of the ZO-1 protein. ZO-1, a marker of gap junctions, is typically expressed in epithelial and endothelial cells (Tornavaca *et al.*, 2015) (Supplementary Fig. S3). Further identification of cell types in the Silk-Ovarioids model was achieved by RNA-FISH and immunodetection in both cortex and medulla. The main ovarian primary cell populations, i.e. stroma, granulosa, endothelial, and perivascular cells, were identified at both mRNA and protein levels. The long-term culture of Silk-Ovarioids points toward the feasible use of Biosilk regarding long-term biocompatibility and support of heterogeneity of the seeded somatic cells up to 42 days of floating culture. This aspect was positively evaluated also in other papers that use Biosilk for organoid culture, including both stem and primary cells (Johansson *et al.*, 2019; Åstrand *et al.*, 2020; Sozzi *et al.*, 2022). Despite the minimal contribution of granulosa cells to the total cellular composition of the Silk-Ovarioids, the presence of this pivotal cell type was confirmed by the staining of markers at mRNA and protein levels and by the low but still detectable secretion of estrone and 17β-estradiol in the culture medium after long-term culture. The functionality of the seeded cells was corroborated by the production of ECM. Based on our RNA-seq data, we focused on the highly expressed proteins, namely collagen type I chain α1 and laminin subunit α1, which are known to be involved in the somatic support of follicles (Fan *et al.*, 2019). Overall, the quality check of the newly established Silk-Ovarioids presents a compact 3D structure in which cells are functional and produce *de novo* ECM.

The pro-angiogenic environment could be mainly initiated and perpetuated by endothelial cells in the Silk-Ovarioids model. In fact, the 3D environment allows endothelial cells to grow, exert their functions, and remodel the ECM to form vessel-like structures, as described in our study. Consistent with our results, successful co-culture of multiple cell types and the formation of micro-vessels have been shown in other silk-based models using both human- and mouse-derived material (Johansson *et al.*, 2019). This underscores the important role of vascularization in 3D cultures to ensure oxygen and nutrient exchange in the inner core (Loh and Choong, 2013), as well as in ovarian 3D models. In a bioprosthetic ovary created using mouse primary cells, the microporous bio-printed scaffold allowed the growth of mouse cells, the attachment of follicles, and its vascularization after grafting back to the mouse (Laronda *et al.*, 2017). This process was vital not only for the survival of the grafts *per se* but also for the growth of the seeded follicles as it promoted the exchange of necessary oxygen, hormones, and nutrients for *in vivo* folliculogenesis post-transplantation (Laronda *et al.*, 2017). Additionally, several studies highlighted the importance of pro-angiogenic factors (e.g. VEGF, estrogen metabolites) (Henríquez *et al.*, 2020; Guzmán *et al.*, 2023) and cytokines (e.g. leukemia inhibitory factor, fibroblast growth factor, IL-6 and 8, and transforming growth factor β family) (Adamczak *et al.*, 2021) in ovarian follicle activation, growth, and development. For instance, the downregulation of estrogen metabolites and VEGF, reported in women diagnosed with polycystic ovarian syndrome, caused the arrest of follicular growth (Henríquez *et al.*, 2020). The secretion of pro-angiogenic cytokines (i.e. IL-6 and IL-8) by the Silk-Ovarioids and the increased expression of *VEGFA* at the transcriptomic level are in line with the activation of angiogenic mechanisms and the presence of endothelial cell-derived tubular structures. Moreover, VEGFA, together with the hypoxia-induced STC-1, a factor involved in follicular angiogenesis as outlined in pigs (Basini *et al.*, 2009) and by our gene-concept hubs, is likely to establish a favorable environment for follicle activation and growth. Indeed, a recent review outlined the putative role of STC-1 in the female reproductive system of mammals, including its involvement in ovarian functions connected to insulin-like growth factor activity, steroidogenesis, and ovulation (Bishop *et al.*, 2021).

In conclusion, this study demonstrates for the first time that the use of Silk-based scaffolds facilitates the successful formation of a human 3D ovarian model that can survive long-term culture without undergoing necrosis or apoptosis, while maintaining healthy cells across the entire structure. This first step could pave the way for establishing a stable and functional ovarian model that can be exploited for numerous applications, ranging from pharmacology and toxicology to basic biology research and clinical purposes. Our Silk-Ovarioids could be utilized as the first ovarian angiogenesis *in vitro* model, as well as a platform for *in vitro* folliculogenesis and clinical applications, due to the low-immunogenic and xeno-free characteristics of the silk scaffold. Once optimized, patient-specific Silk-Ovarioids could serve as a somatic niche to support early follicle growth in young women undergoing gonadotoxic treatments, providing a novel strategy for fertility preservation. Ongoing efforts aim to implement and improve the Silk-Ovarioids model, including the refinement of the selection of relevant cell types and their proportions at the time of seeding. In addition, the introduction of growth factors to the culture media (e.g. midkines and semaphorins) will be tested to assess their potential effects on both angiogenic mechanisms and follicle support and growth, as previously suggested (Rooda *et al.*, 2024). Moreover, the coating of Biosilk with different laminins (i.e. laminin 221, laminin 521, laminin 121) is being evaluated for their ability to support somatic cells attachment and division, along with follicular growth. Currently, folliculogenesis within the Silk-Ovarioids system is under development, and further optimization is required to support follicle survival and growth. Finally, although Biosilk is recognized as a non-immunogenic and biodegradable material, thus suitable for clinical use, future *in vivo* studies should be performed to assess the immunogenic response of the Silk-Ovarioids after xeno-transplantation.

Although many 3D ovarian models are available, including follicle culture using alginate beads, PEGylated fibrin-based structures, and *ex vivo* ovarian cortical tissue culture, each has its advantages and limitations when comparing them to Silk-Ovarioids. While these systems incorporate follicles, they require further optimization to sustain long-term culture, which is essential for folliculogenesis. Additionally, follicle culture systems typically lack the somatic cell components necessary to support *de novo* angiogenesis. In contrast, Silk-Ovarioids establish a somatic niche that enables angiogenesis which, once optimized, provides proper nutrition and growth factors, to support follicle growth over a time frame that is required for the physiological and full-length folliculogenesis.

One limitation of this study is that the ovarian biopsies were collected from patients undergoing androgen treatment. This raises the question of whether androgen exposure may influence the behavior of the cells in Silk-Ovarioids compared to those retrieved from patients not exposed by androgens, or those who have undergone chemo- or radiotherapy. Therefore, further characterization and protocol optimization are needed to better understand this preliminary model and support its future application across diverse patient groups. To create a xeno-free and defined environment for the growth of Silk-Ovarioids, FBS could be replaced by human serum albumin in the culture medium. Finally, the response of the Silk-Ovarioids to hormonal stimulation, such as follicle-stimulating hormone, which is necessary for follicle growth, needs to be tested. This will be addressed in future studies by incorporating follicles into the developing Silk-Ovarioids to test their ability to support *in vitro* follicle growth and development. Additionally, it will be important to test the functionality of the novel capillary formation in the Silk-Ovarioids, for example by further stimulating their formation through the addition of growth factors as VEGF and FGF, and by assessing vascular integration through xenotransplantation in animal models. Overall, Silk-Ovarioids open new avenues for the development of human-based artificial ovaries. This will broaden the possibilities for advancing our knowledge of ovarian pathophysiology and toxicology, and translating ovary models into clinical applications, as well as chemical and pharmaceutical risk assessment.

## Supplementary Material

hoaf042_Supplementary_Data

## Data Availability

RNA-sequencing count matrix is deposited in Gene Expression Omnibus with accession number GSE253571. Raw data are deposited in Swedish National Data Service with the DOI https://doi.org/10.48723/h8cm-bs19. Single-cell RNA-seq data were downloaded from the ArrayExpress database at EMBL-EBI with the accession codes ‘E-MTAb − 8381’ (Wagner *et al.*, 2020). The mass spectrometry proteomics data have been deposited to the ProteomeXchange Consortium via the PRIDE (Perez-Riverol *et al.*, 2022) partner repository with the dataset identifier PXD048710. The code used for the analysis can be found in https://github.com/tialiv/Silk-Ovarioid_project.
