# Supplementary material for: Silk-Ovarioids: establishment and characterization of a human ovarian primary cell 3D-model system
Source: Hum Reprod Open. 2025 Jul 10;2025(3):hoaf042. doi: 10.1093/hropen/hoaf042 (PMC12343022; doi:10.1093/hropen/hoaf042)
Supplement: hoaf042_Supplementary_Data [file hoaf042_supplementary_data.zip › Di_Nisio_et_al_-_Supplementary_Files_legends.docx]

Supplementary Files legends

**Silk-Ovarioids: establishment and characterization of a human ovarian primary cell 3D-model system**

Di Nisio *et al.*

*Corresponding authors. Email: [valentina.di.nisio@ki.se](mailto:valentina.di.nisio@ki.se); [andres.salumets@ki.se](mailto:andres.salumets@ki.se)

Supplementary Figure S1. Expression of DNA damage and apoptotic markers in Silk-Ovarioids. Immunofluorescence staining of γ-H2A.X and Cleaved Caspase 3 in C-Silk-Ov (n=5) and M-Silk-Ov (n=5). Arrows of different colors indicate γ-H2A.X signal (yellow), Cleaved Caspase 3 (red) or the colocalization of the two markers (orange). In the ROIs are evidenced the positive spots for γ-H2A.X and Cleaved Caspase 3. Scale bar for large image represents 200 µm while in inserts scale bar indicates 50 µm. C-Silk-Ov, Cortex-derived Silk-Ovarioid; M-Silk-Ov, Medulla-derived Silk-Ovarioid.

Supplementary Figure S2. Representative images of IgG negative controls and TUNEL positive control for Silk-Ovarioids and tissue immunofluorescence staining. Scale bar represents 200 µm for Silk-Ovarioids and 100 µm for tissue.

Supplementary Figure S3. Cell type specific markers and ZO-1 localization in ovarian tissue as positive control. Immunofluorescence staining of cell specific markers (AMHR2, granulosa cells; PDGFRα, stromal cells; CLDN5 and GPIHBP1, endothelial cells; Cx37 and MCAM, perivascular cells) and gap junction protein ZO-1 in an ovarian cortex cross-section. Scale bar represents 50 µm. AMHR2, anti-Mullerian hormone receptor 2; CLDN5, claudin 5; Cx37, connexin 37; GPIHBP1, Glycosylphosphatidylinositol-anchored high-density lipoprotein-binding protein 1; MCAM, melanoma cell adhesion molecule; PDGFRα, platelet-derived growth factor receptor α; ZO-1, zona occludens 1.

Supplementary Figure S4. Divergence of gene expression in different culture models. (a) Number of DEGs in each comparison in cortex and medulla and the applied cutoff. FDR, abs Log_2_FC, and average expression (as baseMean) cutoff is showed. (b) Heatmap of top 500 variable gene expression in tissue (n=5), 2D (n=4), and Silk-Ov (n=5 for cortex and n=6 for medulla) samples in cortex and medulla. One Silk-Ov sample from cortex was removed due to low library size. Counts were normalized using DESeq2 normalization and scaled to obtain mean equals 0 and standard deviation equals 1. Abs Log2FC, absolute log_2_ fold change; DEGs, differentially expressed genes; FDR, false discovery rate; Silk-Ov, Silk-Ovarioids.

Supplementary Figure S5. Expression of the selected ECM markers, collagen type I chain α1 (Col1α1) and laminin subunit α1 (Lamα1) in tissues (cortex and medulla), 2D monolayer cells (cortex and medulla derived) and Silk-Ovarioids (cortex and medulla derived). C-Silk-Ov, Cortex-derived Silk-Ovarioid; M-Silk-Ov, Medulla-derived Silk-Ovarioids.

Supplementary Figure S6. Heatmap of average z-score of selected genes related to steroidogenesis in cortex and medulla tissue and Silk-Ov samples. Genes were clustered using k-means clustering. Counts were normalized using DESeq2 normalization and scaled to obtain mean equals 0 and standard deviation equals 1. The final gene expression was represented using Z-score. Silk-Ov, Silk-Ovarioids.

Supplementary Table S1. 3D models formation success percentages. *Abbreviations:* 3LGS, 3-layer gradient system; MFOS, matrix-free ovarian spheroids.

Supplementary Table S2. DEGs in tissue vs 2D vs Silk-Ovarioids

Supplementary Table S3. Hypoxia clusters GO over-representation analysis

Supplementary Table S4. Angiogenesis clusters GO over-representation analysis

Supplementary Table S5. GO enrichment analysis on significant DEGs

Supplementary Table S6. Primary and secondary antibodies details

*Abbreviations:* AMHR2, Anti-Mullerian hormone receptor 2; BMP2, Bone morphogenetic protein 2; CLDN5, claudin 5; Col1ɑ1, Collagen type 1 α1; Cx37, Connexin 37; GPIHBP1, glycosylphosphatidylinositol-anchored high-density lipoprotein-binding protein 1; Lamα1, Laminin subunit α1; MCAM, melanoma cell adhesion molecule; MMP2, Matrix metallopeptidase 2; PDGFα, Platelet-derived growth factor α; PDGFRα, Platelet-derived growth factor receptor α; PDGFRβ, Platelet-derived growth factor receptor β; TGFBR2, Transforming growth factor-β receptor type 2; ZO-1, zona occludens 1.

Supplementary Table S7. Steroids detection and measurements in culture media of Silk-Ovarioids

In the second line of the table are reported the LOQs (ng/mL, in plasma) for each specific steroid targeted during the detection and measurement in culture media of Silk-Ovarioids. The red numbers within the table indicate that the steroid was identified and measured, but the level for a reliable quantification is below the LOQ values.

*Abbreviations:* ASD, Androstenedione; COS, Corticosterone; COR, Cortisol; CSO, Cortex-derived Silk-Ovarioid; DHEA, Dehydroepiandrosterone; DeCOR, Deoxycortisol; DHT, Dihydrotestosterone; ETE, Epitestosterone; HCOR, Hydroxycortisol; HPR, Hydroxyprogesterone; LOQ, limit of quantification; MSO, Medulla-derived Silk-Ovarioid; PRE, Pregnenolone; PRO, Progesterone; Pt: patient; TES, Testosterone, ALDO, Aldosterone; E1, Estrone; E2, β-Estradiol.
