# Supplementary material for: Silk-Ovarioids: establishment and characterization of a human ovarian primary cell 3D-model system
Source: Hum Reprod Open. 2025 Jul 10;2025(3):hoaf042. doi: 10.1093/hropen/hoaf042 (PMC12343022; doi:10.1093/hropen/hoaf042)
Supplement: hoaf042_Supplementary_Data [file hoaf042_supplementary_data.zip › Fig._S1_EO.pdf]

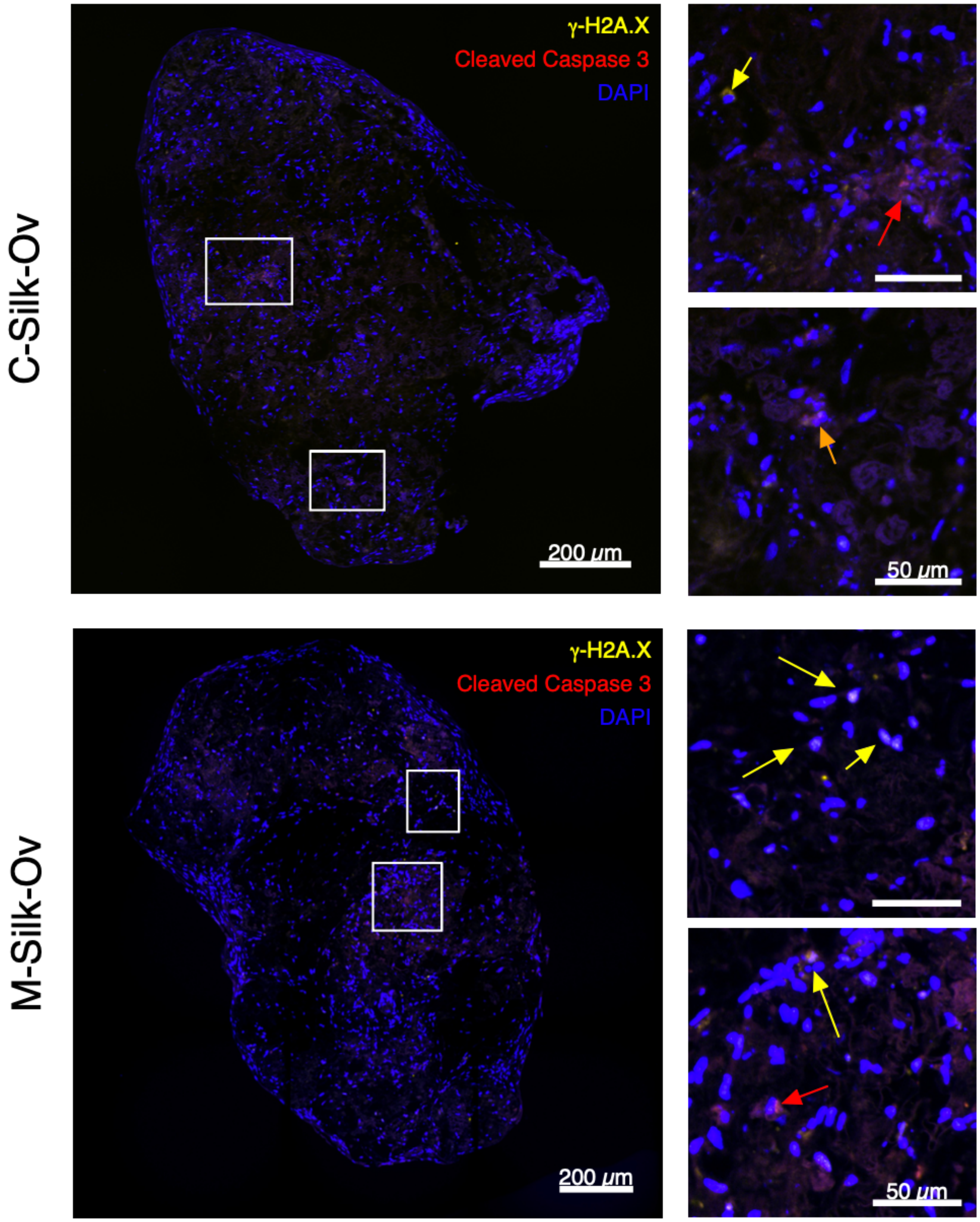

Supplementary Fig. S1. Expression of DNA damage and apoptotic markers in Silk-Ovarioids

Immunofluorescence staining of γ-H2A.X and Cleaved Caspase 3 in C-Silk-Ov (n=5) and M-Silk-Ov (n=5). Arrows of different colors indicate γ-H2A.X signal (yellow), Cleaved Caspase 3 (red) or the colocalization of the two markers (orange). In the ROIs are evidenced the positive spots for γ-H2A.X and Cleaved Caspase 3. Scale bar for large image represents 200 µm while in inserts scale bar indicates 50 µm. C-Silk-Ov, Cortex-derived Silk-Ovarioid; M-Silk-Ov, Medulla-derived Silk-Ovarioid.
