# Supplementary material for: Silk-Ovarioids: establishment and characterization of a human ovarian primary cell 3D-model system
Source: Hum Reprod Open. 2025 Jul 10;2025(3):hoaf042. doi: 10.1093/hropen/hoaf042 (PMC12343022; doi:10.1093/hropen/hoaf042)
Supplement: hoaf042_Supplementary_Data [file hoaf042_supplementary_data.zip › Fig._S2_EO.pdf]

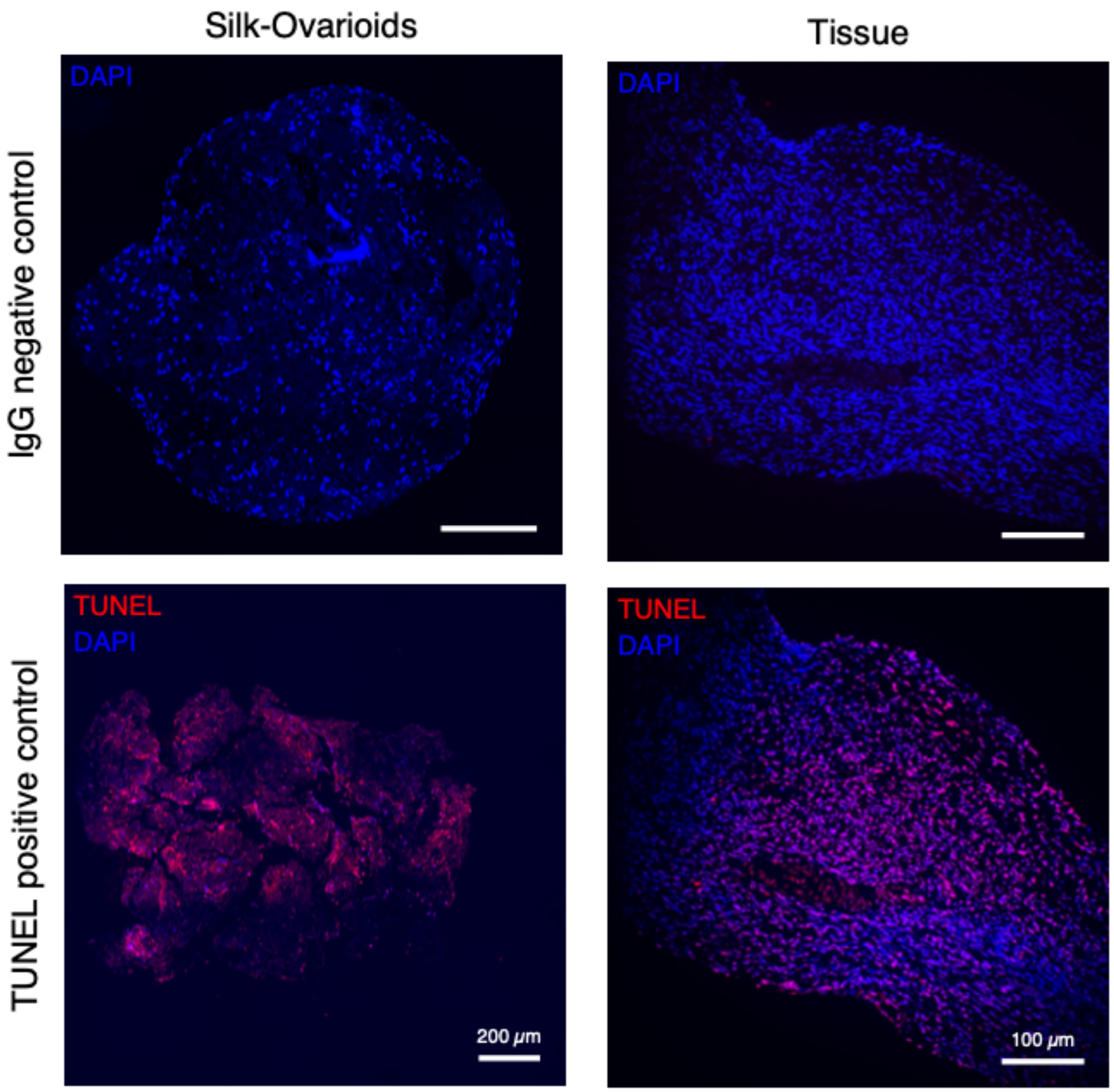

Supplementary Fig. S2. Representative images of IgG negative controls and TUNEL positive control for Silk-Ovarioids and tissue immunofluorescence staining. Scale bar represents 200 µm for Silk-Ovarioids and 100 µm for tissue.
