# Supplementary material for: Silk-Ovarioids: establishment and characterization of a human ovarian primary cell 3D-model system
Source: Hum Reprod Open. 2025 Jul 10;2025(3):hoaf042. doi: 10.1093/hropen/hoaf042 (PMC12343022; doi:10.1093/hropen/hoaf042)
Supplement: hoaf042_Supplementary_Data [file hoaf042_supplementary_data.zip › Fig._S3_EO.pdf]

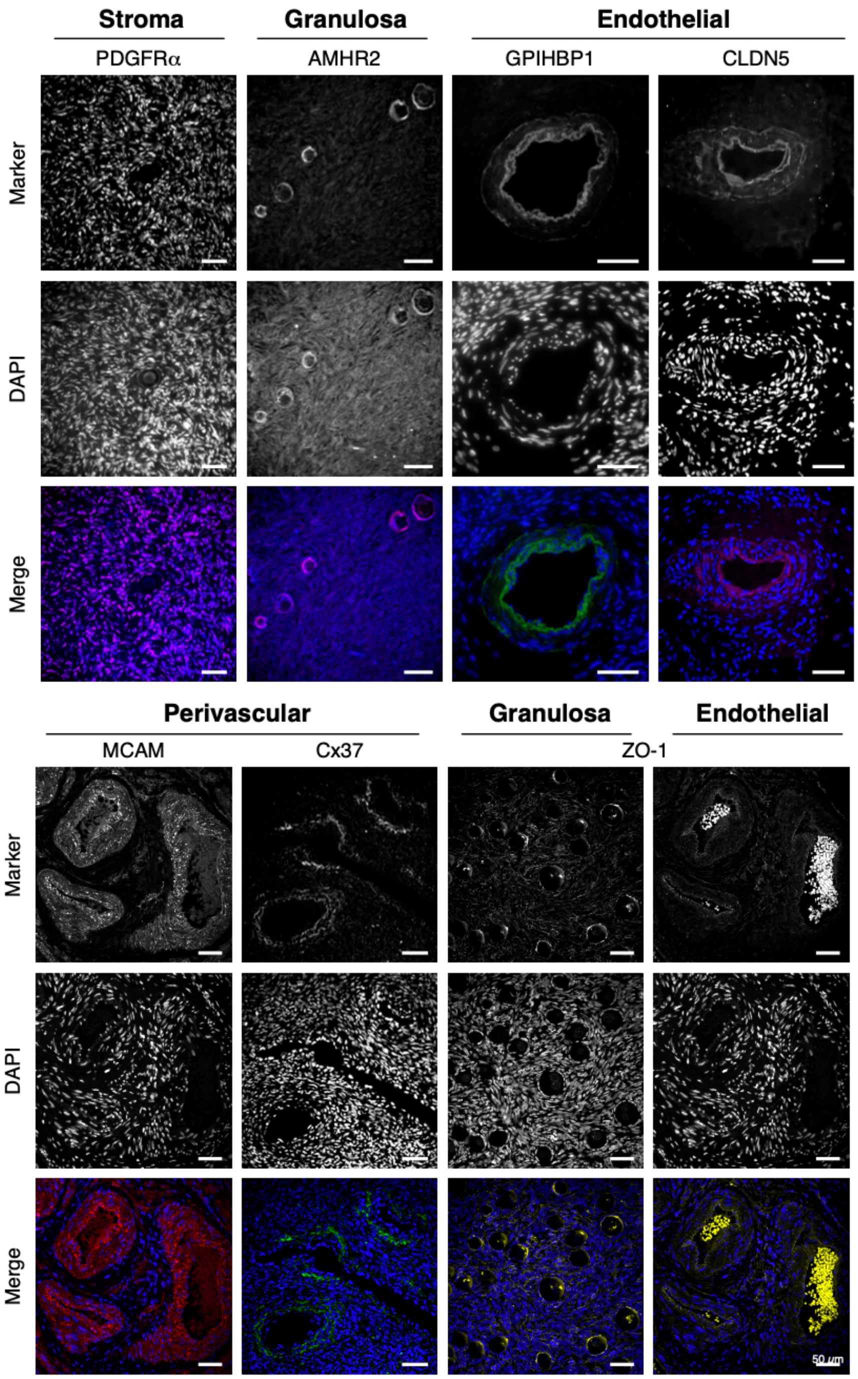

Supplementary Fig. S3. Cell type specific markers and ZO-1 localization in ovarian tissue as positive control.

Immunofluorescence staining of cell specific markers (AMHR2, granulosa cells; PDGFRα, stromal cells; CLDN5 and GPIHBP1, endothelial cells; Cx37 and MCAM, perivascular cells) and gap junction protein ZO-1 in an ovarian cortex cross-section. Scale bar represents 50 µm. AMHR2, anti-Mullerian hormone receptor 2; CLDN5, claudin 5; Cx37, connexin 37; GPIHBP1, Glycosylphosphatidylinositol-anchored high-density lipoprotein-binding protein 1; MCAM, melanoma cell adhesion molecule; PDGFRα, platelet-derived growth factor receptor α; ZO-1, zona occludens 1.
