# Supplementary material for: Silk-Ovarioids: establishment and characterization of a human ovarian primary cell 3D-model system
Source: Hum Reprod Open. 2025 Jul 10;2025(3):hoaf042. doi: 10.1093/hropen/hoaf042 (PMC12343022; doi:10.1093/hropen/hoaf042)
Supplement: hoaf042_Supplementary_Data [file hoaf042_supplementary_data.zip › Fig._S4_EO.pdf]

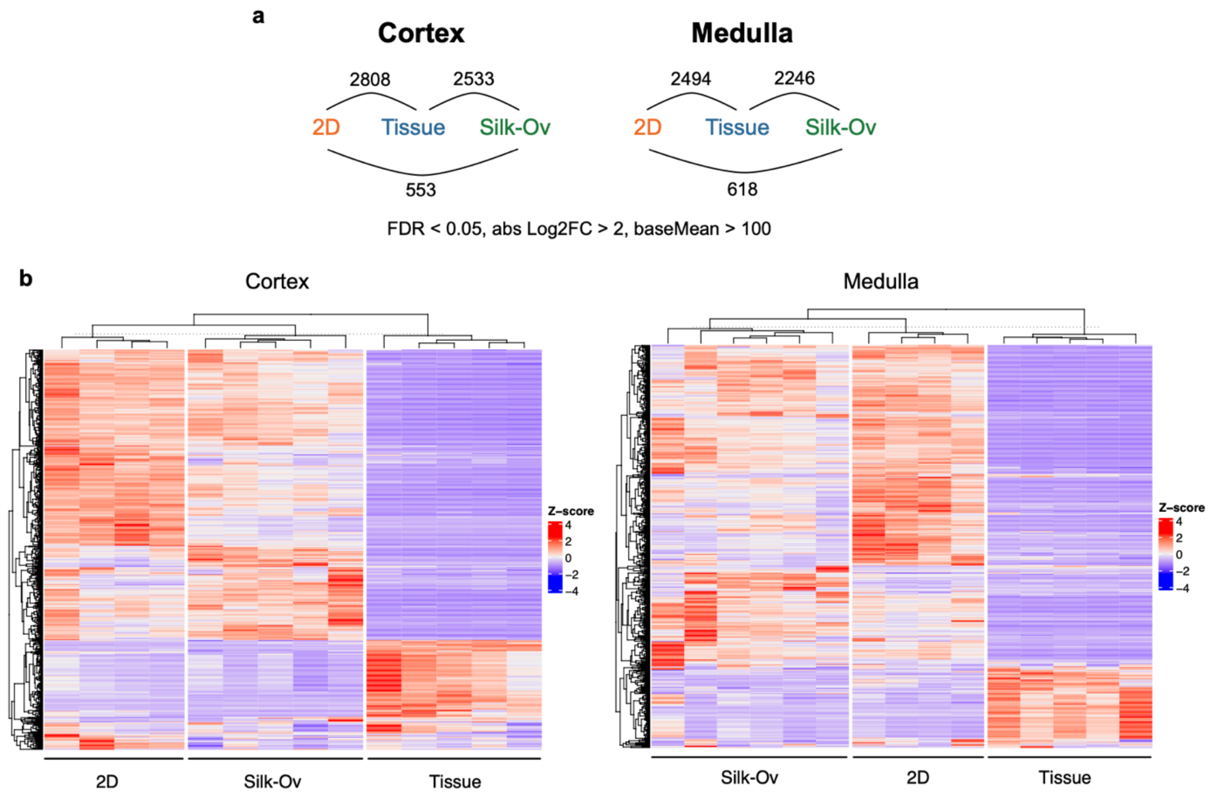

Supplementary Fig. S4. Divergence of gene expression in different culture models.

(**a**) Number of DEGs in each comparison in cortex and medulla and the applied cutoff. FDR, abs Log_2FC, and average expression (as baseMean) cutoff is showed. (**b**) Heatmap of top 500 variable gene expression in tissue (n=5), 2D (n=4), and Silk-Ov (n=5 for cortex and n=6 for medulla) samples in cortex and medulla. One Silk-Ov sample from cortex was removed due to low library size. Counts were normalized using DESeq2 normalization and scaled to obtain mean equals 0 and standard deviation equals 1. Abs Log2FC, absolute $\log_2$ fold change; DEGs, differentially expressed genes; FDR, false discovery rate; Silk-Ov, Silk-Ovarioids.
