# Supplementary material for: Silk-Ovarioids: establishment and characterization of a human ovarian primary cell 3D-model system
Source: Hum Reprod Open. 2025 Jul 10;2025(3):hoaf042. doi: 10.1093/hropen/hoaf042 (PMC12343022; doi:10.1093/hropen/hoaf042)
Supplement: hoaf042_Supplementary_Data [file hoaf042_supplementary_data.zip › Fig._S5_EO.pdf]

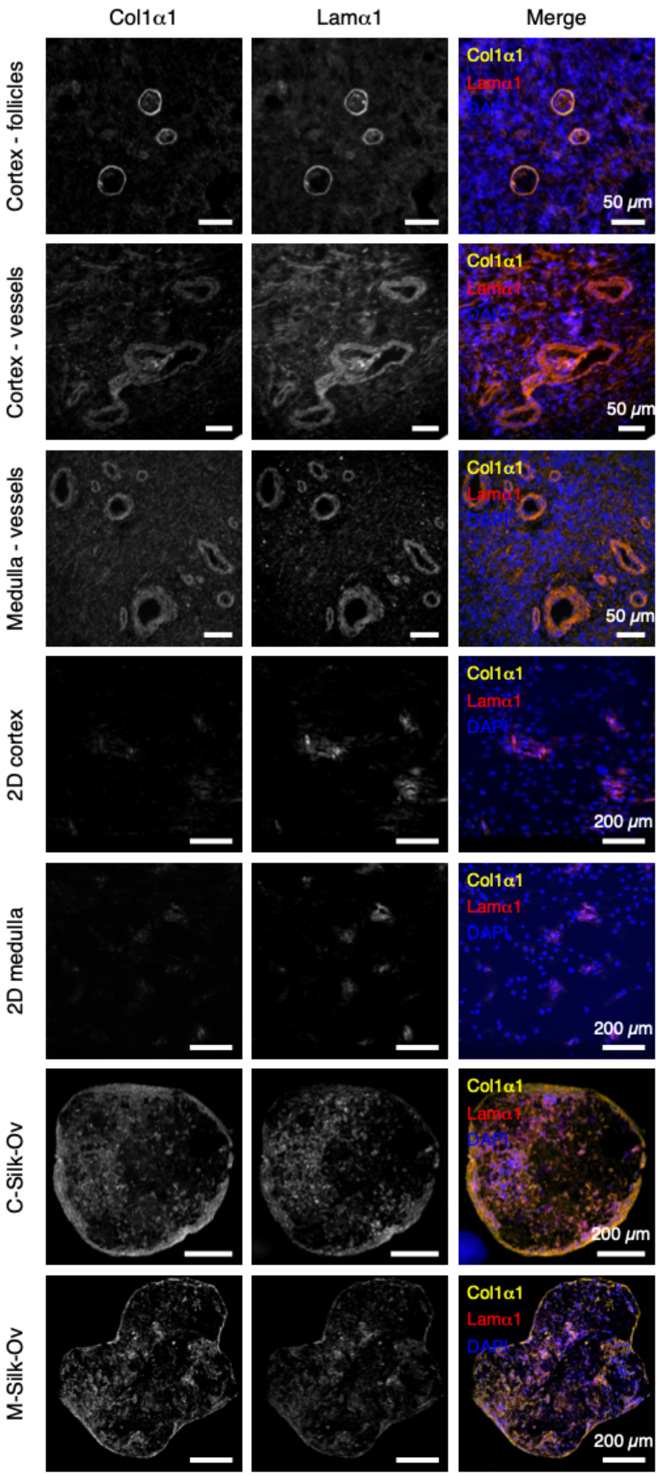

Supplementary Fig. S5. Expression of the selected ECM markers, collagen type I chain α1 (Col1α1) and laminin subunit α1 (Lamα1) in tissues (cortex and medulla), 2D monolayer cells (cortex and medulla derived) and Silk-Ovarioids (cortex and medulla derived). C-Silk-Ov, Cortex-derived Silk-Ovarioid; M-Silk-Ov, Medulla-derived Silk-Ovarioids.
