# Supplementary material for: Silk-Ovarioids: establishment and characterization of a human ovarian primary cell 3D-model system
Source: Hum Reprod Open. 2025 Jul 10;2025(3):hoaf042. doi: 10.1093/hropen/hoaf042 (PMC12343022; doi:10.1093/hropen/hoaf042)
Supplement: hoaf042_Supplementary_Data [file hoaf042_supplementary_data.zip › Fig._S6_EO.pdf]

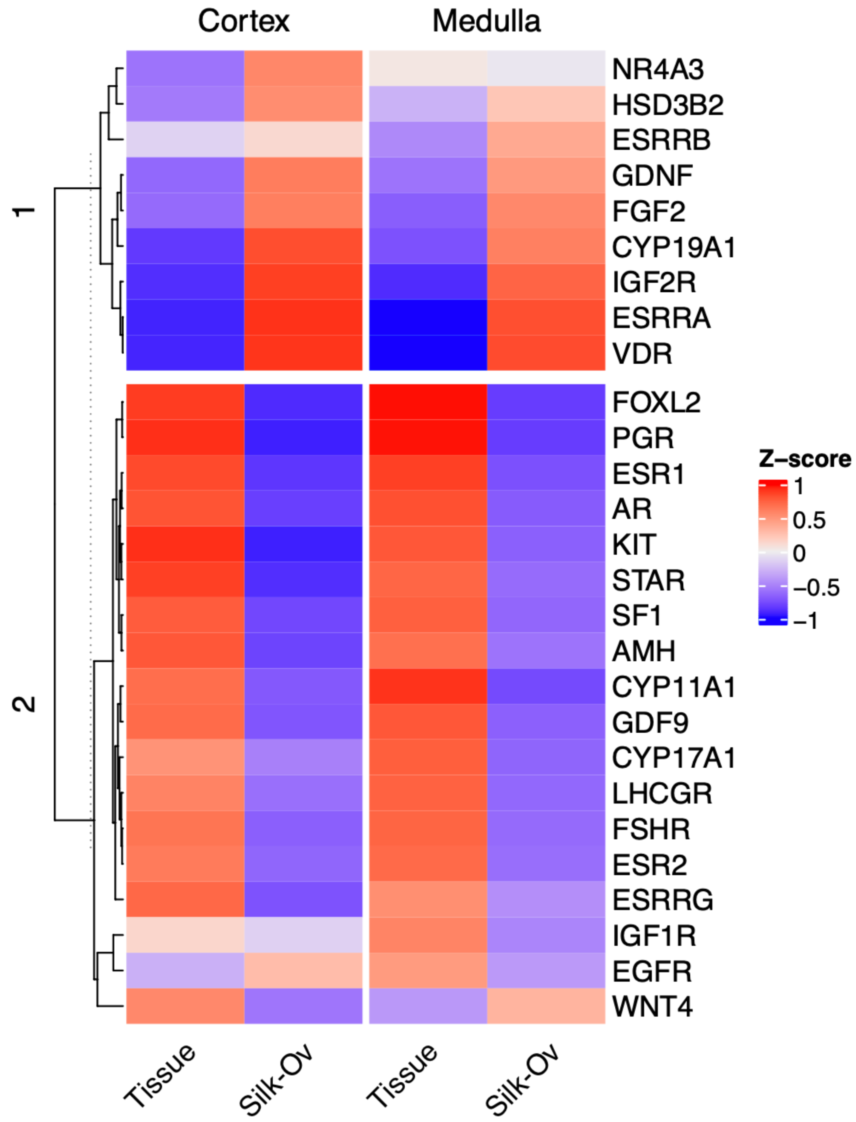

Supplementary Fig. S6. Heatmap of average z-score of selected genes related to steroidogenesis in cortex and medulla tissue and Silk-Ov samples.

Genes were clustered using k-means clustering. Counts were normalized using DESeq2 normalization and scaled to obtain mean equals 0 and standard deviation equals 1. The final gene expression was represented using Z-score. Silk-Ov, Silk-Ovarioids.
